# Supplementary material for: An adenoviral-vectored vaccine protects mice against aerosol challenge with Yersinia pestis
Source: Mol Ther. 2026 Feb 24;34(6):3518–34. doi: 10.1016/j.ymthe.2026.02.036 (PMC13246311; doi:10.1016/j.ymthe.2026.02.036)
Supplement: Supplementary file 1 [file mmc1.pdf]

YMTHE, Volume *34*

Supplemental Information

An adenoviral-vectored vaccine protects mice against aerosol challenge with *Yersinia pestis*

Christina Dold, Laura Silva-Reyes, Young Chan Kim, Luke Blackwell, Patricia Campos, Aline Linder, Alice Bridges-Webb, Mwila Kasanyinga, Cesar Lopez-Camacho, Amy Challis, Graham Hatch, Susan Fotheringham, Peter T. Beernink, Adrian V. Hill, Simon G.P. Funnell, A.J. Pollard, and C.S. Rollier

Supplemental Materials and Methods

Detection of antigen expression in cell culture

Detection of antigen expression was performed in HeLa cells infected with the antigen-expressing or control vectors as described previously *(28)*, using anti-F1 (Abcam) and V antibodies, and recombinant F1 and V proteins (generous gift from Dr Diane Williamson, DSTL, UK)*(52)*. Western blotting was performed on cell lysate and cell supernatant: Transduced HeLa cells were washed twice with ice-cold PBS and harvested in cold PBS. Cells were lysed in 1X Laemmli buffer. For supernatant, 4X Laemmli buffer was added; both cells and supernatant were boiled at 100°C for five minutes. In addition, a fraction of the supernatant was concentrated using an Amicon Ultra column with a 3kDa cut-off (Millipore, UFC200324. Concentrated samples were prepared in 4X Laemmli buffer and boiled at 100°C. Total extracts and cell supernatants (15ul volume) were resolved by SDS/PAGE and transferred to PVDF membranes. Blots were blocked with PBS-0.1% (v/v) Tween plus 5% (v/v) milk and incubated with anti-F1 (Abcam ab20833, anti-F1 antigen antibody mAb [YPF19]) or anti-V antibody (Invitrogen, MA1-23088) for one hour, followed by 5 washes with PBS- 0.1% (v/v) Tween and incubation with HRP-conjugated secondary antibody (1:5000). After final wash, Chemiluminescence (Perkin-Elmer Life Sciences, Boston, MA) was visualized using the BioRad ChemiDoc SRS device.

Splenocyte extraction

After removal, spleens were transferred to gentleMACS C tubes (Miltenyi Biotec) containing 5ml of autoMACS running buffer (Miltenyi Biotec) and dissociated using the spleen program

on the gentleMACS Dissociator (Miltenyi Biotec). After centrifugation at 300g for 10 minutes, the pellets were resuspended in Red Blood Cell Lysis solution (Qiagen) for 5 minutes. The reaction was stopped by the addition of 10ml of AutoMACS Rinsing solution (Miltenyi Biotec). After a second centrifugation at 300g for 10 minutes, cells were filtered through 70µm sieve into sterile 50ml tubes and centrifuged again. Cells were then resuspended in 10ml of DMEM+10%FBS (Sigma) and counted using a Muse Cell Analyzer counter (Luminex). Concentration was then adjusted at 4 x 106 cells/ml in DMEM+10%FBS for T cell fluorospots.

Lung cell extraction

After removal, lungs were transferred to gentleMACS C tubes containing 5ml of digestion medium (DMEM+10%FBS with 30µg/ml DNAse (Sigma) and 0.7µg/ml Collagenase (Sigma)). Lungs were kept on ice until dissociation with the gentleMACS Dissociator (lung_02 program) and then placed in a shaking incubator for 60 minutes at 37°C. Dissociated lungs were then filtered through 100µm sieve into sterile 50ml tubes. After addition of 25ml of cold DMEM+10%FBS, tubes were centrifuged at 300g for 10 minutes. Pellets were resuspended in RBC lysis buffer for 5 minutes maximum and then filtered through a 70µm sieved after addition of 10ml of cold DMEM+10%FBS. After centrifugation, cells were resuspended in 5ml DMEM+10%FBS and counted with the Muse Cell Analyzer counter (Luminex).

Detection and quantification of T-cell responses

IFN-γ and IL-5 producing T cells were quantified by using a mouse FluoroSpot kit (Mabtech, FSP-4144-10). After activation with 15 µl 35% Ethanol and 5 washes with sterile water, plates

were coated with anti-IFNγ and anti-IL-5 monoclonal capture antibodies diluted in sterile PBS at 15 μg/ml and 10 μg/ml, respectively. Plates were then sealed and kept overnight at 4 °C. The following day, plates were washed 5 times with sterile PBS and blocked with DMEM+10%FBS for minimum 30 minutes at room temperature. To ensure efficient and reproducible stimulation of antigen-specific T-cells independent of variability in antigen uptake, processing and presentation, we used overlapping peptide pools spanning the full antigen sequence of the F1 and V proteins. Two peptide pools (15-mer overlapping by 11-amino acids) were diluted in DMEM+10%FBS at 6μg/ml (final concentration after addition of cells:3μg/ml). 50μl of diluted F1 and V peptide pools were added to the wells. DMSO (Sigma) and concanavalin A (Sigma) were used as negative and positive controls for each sample. After being resuspended at 4 x 10^6 cells/ml in DMEM+10%FBS, 50μl of splenocytes or lung cells were added to the peptides wells in triplicates and incubated for 18-20h at 37 °C, 5% CO2, 95% humidity. Detection of spots was undertaken according to the manufacturer's instructions (Mabtech) and analysed with AID ELISpot software 8.0 (Autoimmun Diagnostika).

Supplemental tables:

Table S1. Characteristics of the nine transgene designs. Designs included F1 and V genes inserted into separate or a single vaccine vector, with differences in the bacterial sequence used in addition to a mammalian signal sequence, and different flexible linkers between the two antigens

Transgene	Antigens inserted	Bacterial signal sequence (SS)	Linker
F1 FL[1]	F1	Wild type SS	None
F1 T[2]	F1	No SS	None
F1 fHbpSS[3]	F1	fHbp SS	None
V	V	No SS	None
F1 T (L1) V	F1 + V	No SS	L1 [4]
F1 T (L2) V	F1 + V	No SS	L2 [5]
F1 FL (L1) V	F1 + V	Wild type SS	L1
F1 FL (L2) V	F1 + V	Wild type SS	L2
fHbpSS F1 (L1) V	F1 + V	fHbp SS	L1

[1] FL: full length immature F1 protein, containing its wild type signal sequence

[2] T: truncated mature F1 protein (no bacterial signal sequence)

[3] fHbpSS: meningococcus fHbp signal sequence NRTAFCCLSLTTALILTACSSG

[4] L1: linker used to fuse F1 and V, amino acid sequence ADLPSLAADFVE

[5] L2: linker used to fuse F1 and V, amino acid sequence GGGGSGGGGSGGGGS

Table S2. Number of mouse sera assessed by F1 ELISA at each time point in the longitudinal experiment dataset shown in Figure 2. Groups of 6 or 12 mice were immunized with the vaccines mentioned in the first column. Tail bleeds were collected at 2, 6, 10, 18, 26 and 34 weeks, without exceeding the ethically permitted maximum amount of blood per mouse, per time point and per month. Too many datapoints were missing at week 10. The number of sera collected and assessed by F1 ELISA is indicated in the table, and data shown in Figure 2B to E.

Vaccine group	N	Week 2	Week 18	Week 26	Week 34
Naive	12	8	2	11	9
rF1 + rV proteins	6	6	4	6	5
HuAd5 empty	6	6	5	5	5
HuAd5 F1 FL	6	6	6	6	5
HuAd5 F1 fHbpSS	6	6	6	6	6
HuAd5 F1 T	6	6	6	6	6
HuAd5 F1 FL (L1) V	12	11	11	10	12
HuAd5 F1 FL (L2) V	6	5	6	6	6
HuAd5 F1 fHbpSS (L1) V	6	6	6	6	6
HuAd5 F1 T (L1) V	6	5	6	6	6
HuAd5 F1 T (L2) V	6	6	5	6	6

Table S3. Number of mouse sera assessed by V ELISA at each time point in the longitudinal experiment dataset shown in Figure 2. Groups of 6 or 12 mice were immunized with the vaccines mentioned in the first column. Tail bleeds were collected at 2, 6, 10, 18, 26 and 34 weeks, without exceeding the ethically permitted maximum amount of blood per mouse, per time point and per month. Too many datapoints were missing at week 10, 26 and 34. The

number of sera collected and assessed by V ELISA is indicated in the table, and data shown in Figure 2F and G.

Vaccine group	N	Week 2	Week 18
Naive	12	12	2
rF1 + rV proteins	6	6	5
HuAd5 empty	6	5	4
HuAd5 V	6	6	6
HuAd5 F1 FL (L1) V	12	11	11
HuAd5 F1 FL (L2) V	6	6	4
HuAd5 F1 fHbpSS (L1) V	6	6	5
HuAd5 F1 T (L1) V	6	6	6
HuAd5 F1 T (L2) V	6	6	6

Supplemental Figures:

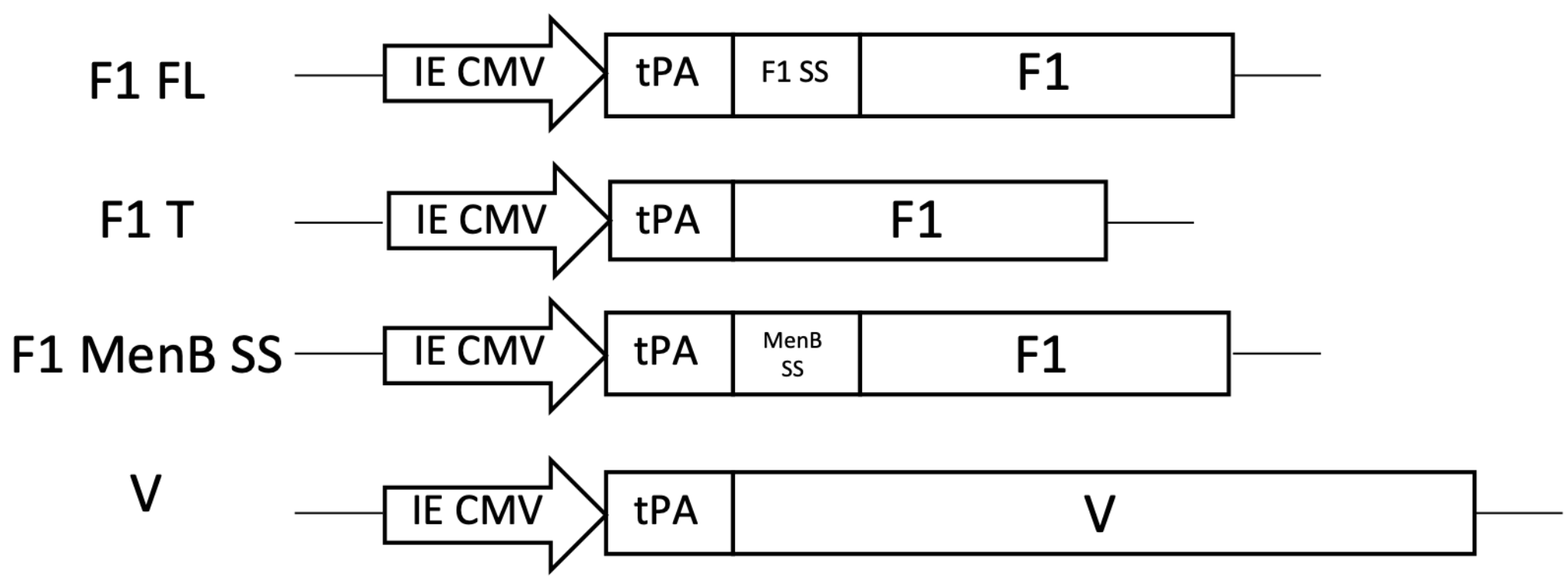

Figure S1. Schematic representation of the four monovalent transgenes, encoding either F1 or V. IE CMV: human cytomegalovirus major immediate early promoter. tPA: Human tissue plasminogen activator (tPA) signal peptide sequence. F1 signal sequence (SS): MKKISSVIAIALFG- TIATANAAD, fHbp signal sequence: NRTAFCCLSLTTALILTACSSG. The transgenes were inserted into the E1 region of the HuAd5 genome.

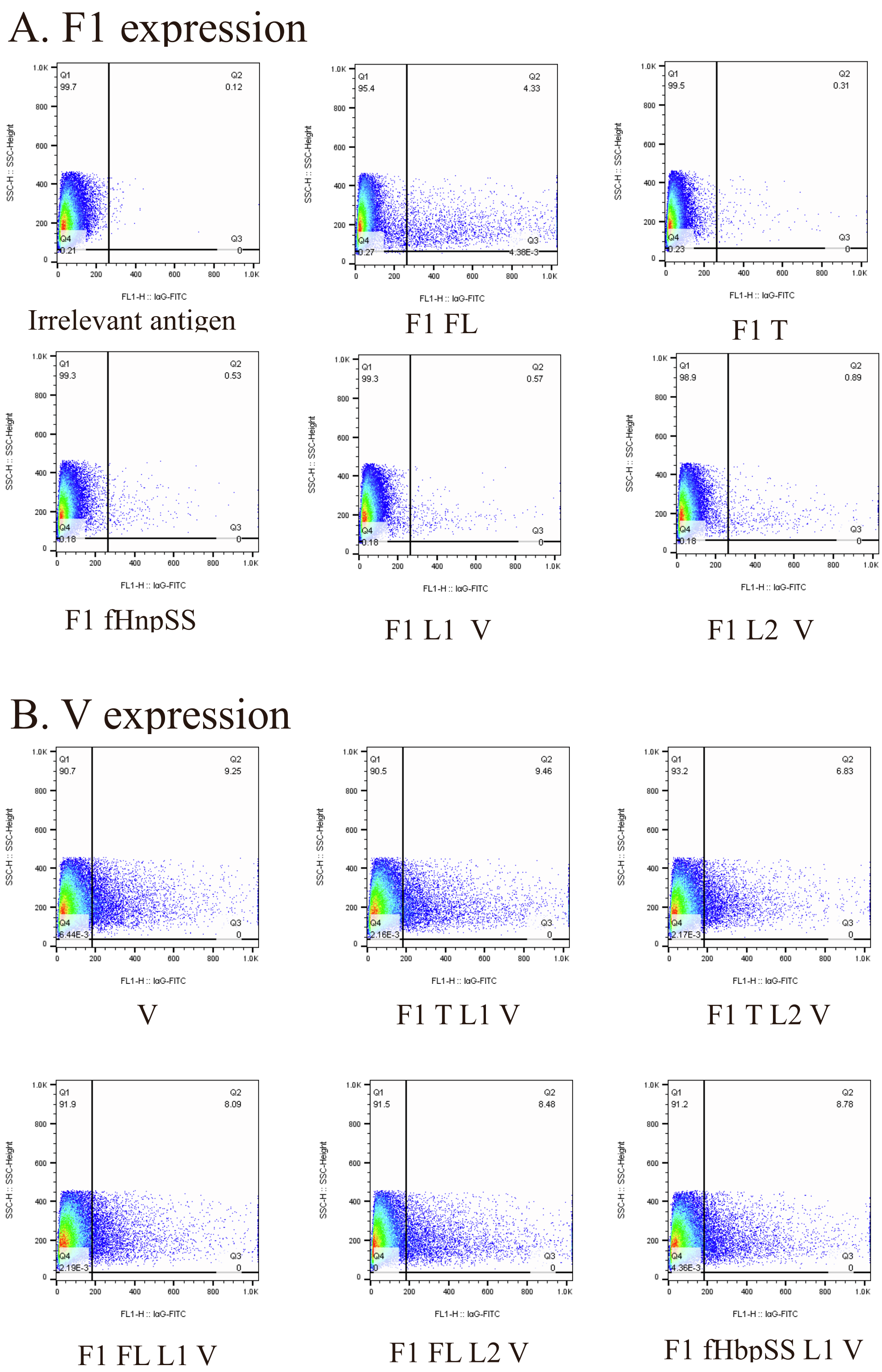

Figure S2. The different monovalent or fusion constructs express F1 (A) and V (B) in HeLa cells. Cells were infected with the HuAd5 Adenoviral vectors encoding the antigens indicated. Detection of F1 and V was performed by flow cytometry using anti-F1 and anti-V

monoclonal antibodies. Panels show the protein expression in viable cells with the constructs indicated under each panel, in one representative experiment. The F1 constructs differed in the antigen bacterial signal sequence used after the tPA signal: either full length F1 bacterial sequence (FL), no bacterial sequence (T) after the tPA, or the F1 bacterial sequence replaced with the group B meningococcal (MenB) fHbp bacterial signal sequence (fHbpSS). The fusion constructs containing both F1 and V were constructed with two different flexible linkers (L1 or L2).

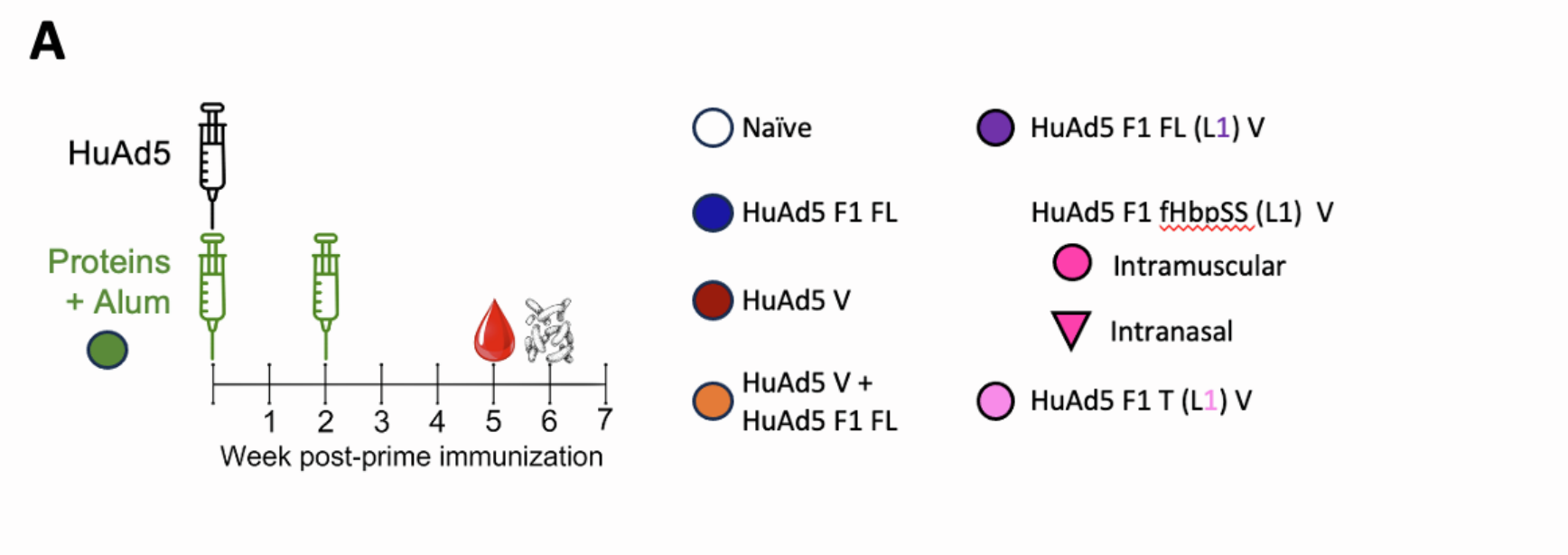

Clinical symptom	score assigned
Healthy	0
Lethargic	1
Ruffled Fur	2
Arched Back	3
Eyes Shut	3
Wasp Waisted	3
Laboured Breathing	5
Immobile	9
Neurological Signs	10
Death in cage	10

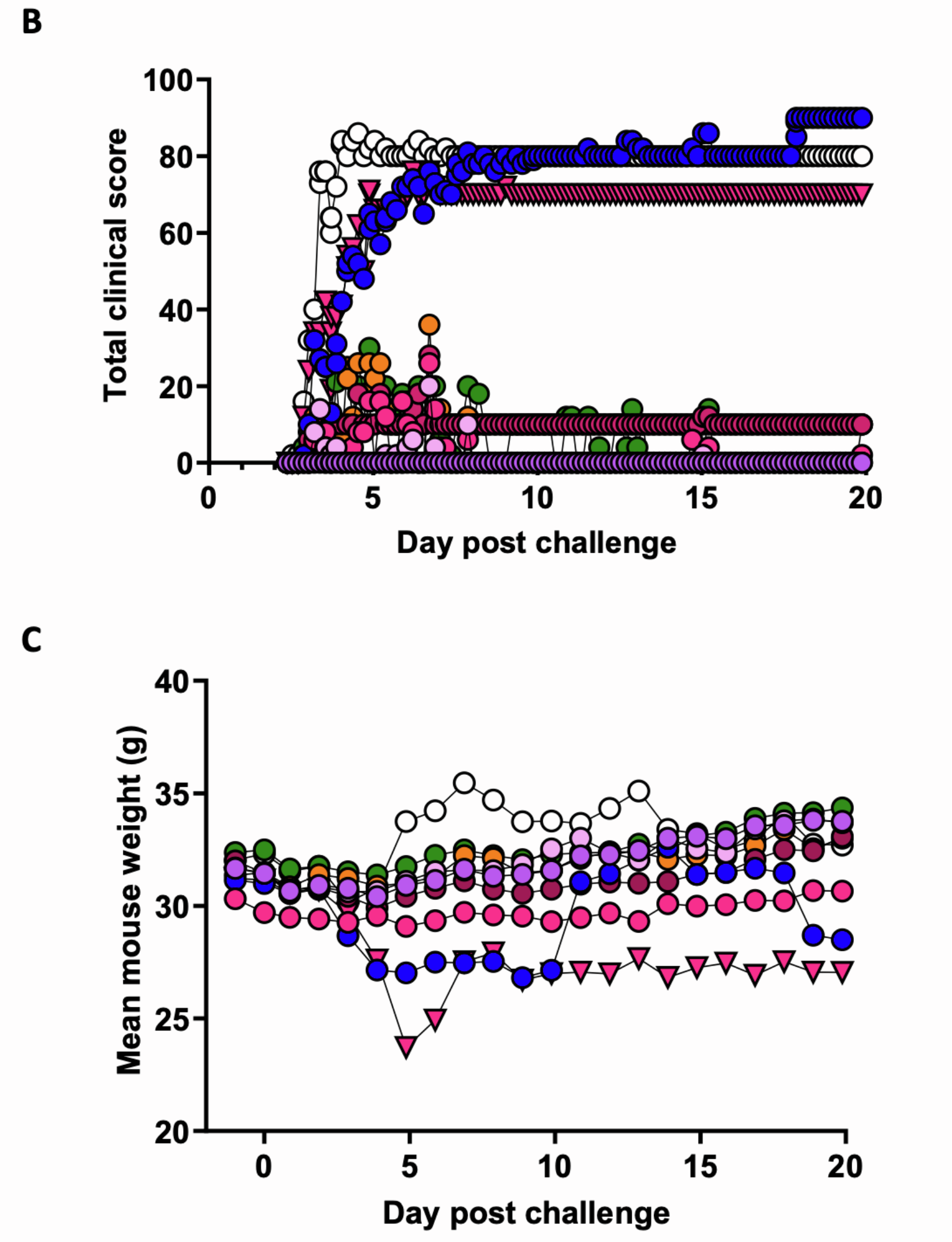

Figure S3. Impact of the different vaccines on clinical scores and mouse weights post challenge in mice. Nine groups of NIH Swiss mice (n=10) were included in the study as described in Figure 4A and panel A. All vaccines were administered by the intramuscular route

except for one group which received HuAd5 F1 fHbp SS (L1) V by the intranasal route One mouse died for unrelated reason in this group, reducing the number to 9 animals. The clinical scores described in (A) were assessed twice a day and represented over time (B). The average group surviving mouse weight over time are shown in panel C.

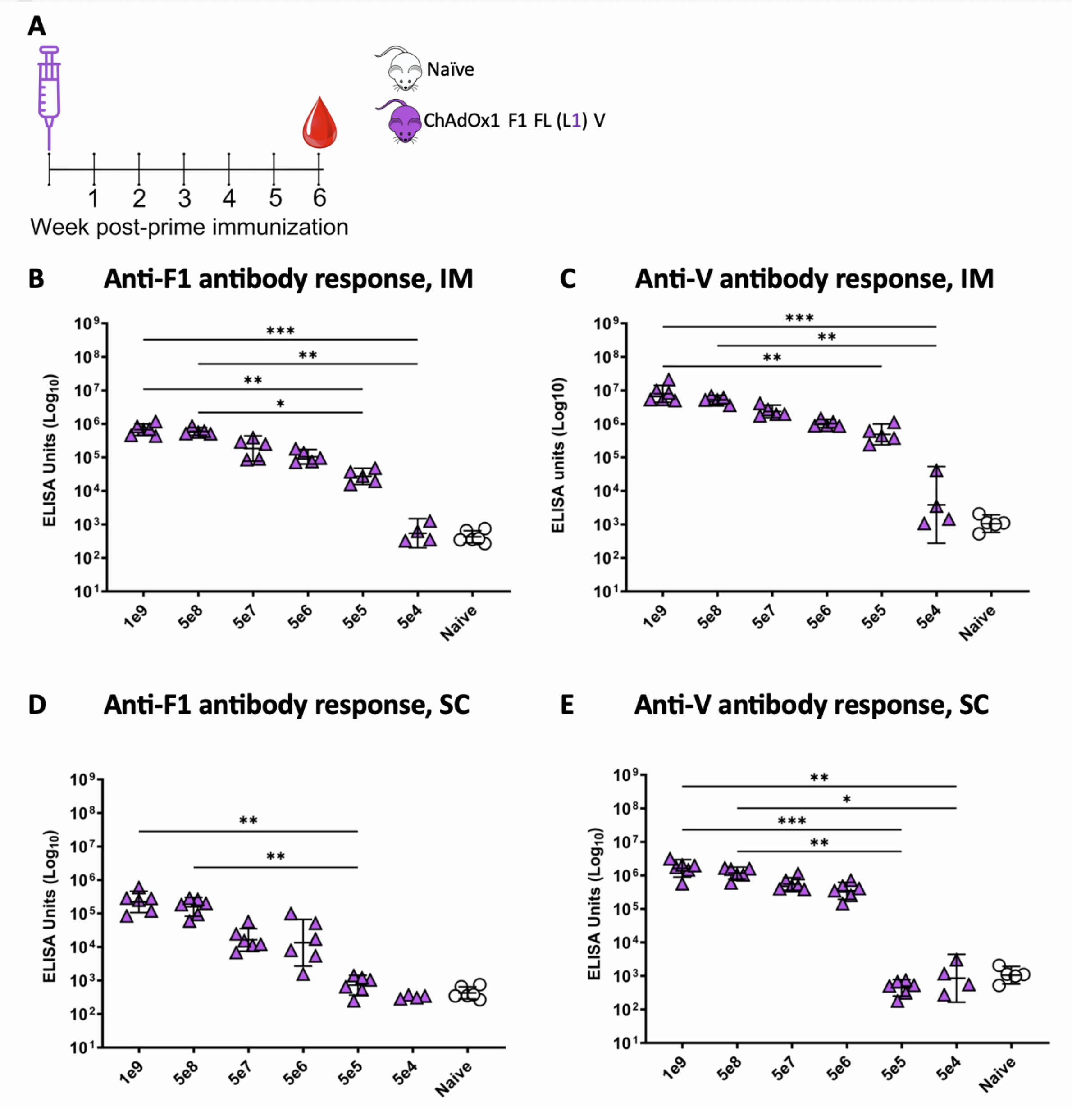

Figure S4. Dose response of the clinically relevant ChadOx1 Plague vaccine candidate given by the intramuscular (IM) and subcutaneous (SC) routes in NIH Swiss mice. Groups of outbred

NIH Swiss mice (n=6) were immunized with a single dose of ChAdOx1 (purple triangles) Plague at the doses indicated in the X axis of each panel ($5x10^4$ to 10^9 infectious units). ChAdOx1 Plague was administered by the intramuscular (IM) route as shown in panels B and C or the subcutaneous (SC) route in panels D and E. The serum IgG to F1 (B) and V (C) respectively were measured at week 6. Shown are individual responses with geometric mean and 95% confidence intervals for each group. Data were analyzed by Kruskal Wallis with Dunns multiple comparison test (* p<0.05, ** p<0.01, *** p<0.001).

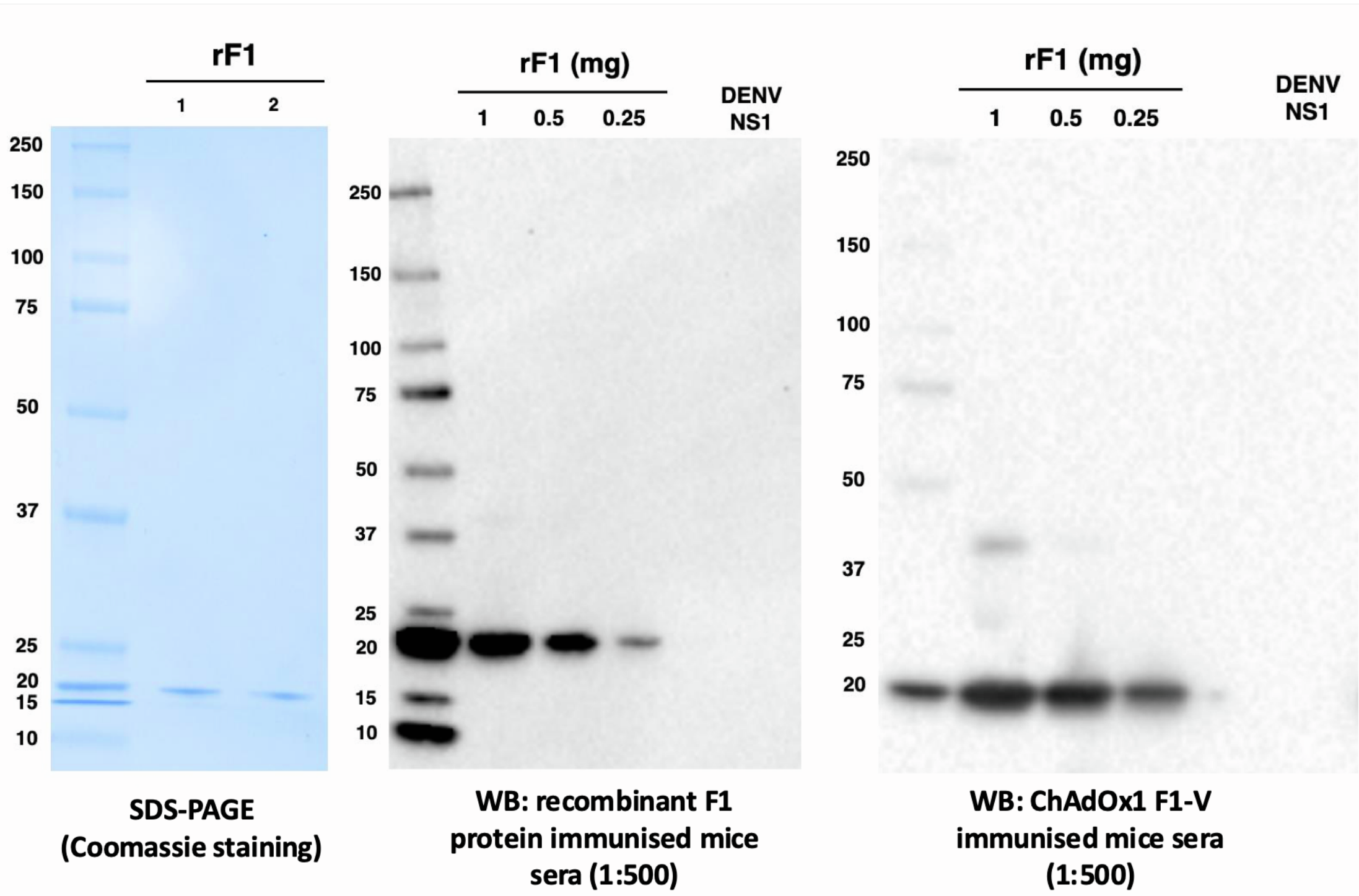

Figure S5. The recombinant F1 protein profile is similar to the original batch. SDS-PAGE (left panel) and western blot (central and right panels) of the recombinant F1 detected with the sera indicated below each panel. rF1 indicates recombinant F1, the number above the lanes indicate the loading doses, DENV NS1 is a negative control (Dengue virus non-structural protein 1), WB: western blot.
