# Supplementary material for: An adenoviral-vectored vaccine protects mice against aerosol challenge with Yersinia pestis
Source: Mol Ther. 2026 Feb 24;34(6):3518–34. doi: 10.1016/j.ymthe.2026.02.036 (PMC13246311; doi:10.1016/j.ymthe.2026.02.036)
Supplement: Supplementary file 2 [file mmc2.pdf]

Molecular Therapy
Original Article

An adenoviral-vectored vaccine protects mice against aerosol challenge with *Yersinia pestis*

Christina Dold,[1,7,8] Laura Silva-Reyes,[1,8] Young Chan Kim,[1,8] Luke Blackwell,[1] Patricia Campos,[1] Aline Linder,[1] Alice Bridges-Webb,[1] Mwila Kasanyinga,[1] Cesar Lopez-Camacho,[2] Amy Challis,[3] Graham Hatch,[3] Susan Fotheringham,[3] Peter T. Beernink,[4] Adrian V. Hill,[2] Simon G.P. Funnell,[3,5,8] A.J. Pollard,[1,8] and C.S. Rollier[1,6,8]

[1]Oxford Vaccine Group, Department of Paediatrics, University of Oxford and the NIHR Oxford Biomedical Research Centre, CCVTM, Churchill Lane, OX37LE Oxford, UK; [2]Jenner Institute, University of Oxford, Old Road Campus Research Building, OX3 7DQ Oxford, UK; [3]United Kingdom Health Security Agency, Manor Farm Road, Porton Down, SP4 0JG Salisbury, UK; [4]Department of Pediatrics, University of California, San Francisco, Oakland, CA, USA; [5]Quadram Institute Bioscience, Norwich Research Park, Norfolk, UK; [6]Faculty of Health and Medical Sciences, School of Biosciences, Department of Biochemistry and Physiology, University of Surrey, Guildford, UK

With the success of viral-vectored vaccines against Ebola and SARS-CoV-2, these platforms are now established as promising tools for biodefence against outbreak pathogens. Here, we report the development of adenoviral-vectored vaccines against plague, caused by *Yersinia pestis*. Replication-deficient adenoviral-vectored constructs expressing the protective *Y. pestis* antigens F1 and LcrV, alone or in combi and combinations thereof were created. Expression of the antigens was confirmed *in vitro*, and immunogenicity assessed in mice. Protective efficacy was evaluated using a virulent *Y. pestis* aerosol challenge model in mice. All vaccine candidates induced high serum IgG antibody responses. Notably, the antibody responses were similar when vectors expressing different antigens were mixed or when using a single vector expressing a chimeric fusion of the two antigens. All of the six experimental vaccine formulations tested via intramuscular injection containing either the V gene or purified V protein provided 90%–100% protection from mortality due to inhalational infection with *Y. pestis*. Notably, only the human adenovirus 5 construct expressing a full length F1-V fusion provided 100% protection from both morbidity and mortality after a single dose. In contrast, naive mice and 50% of mice immunized with two doses of the recombinant proteins with adjuvant displayed clinical signs of illness (morbidity) despite protection from mortality. Importantly, in a follow-up virulent aerosol challenge study, a single dose of ChAdOx1 F1-V fusion provided protection comparable to that achieved with HuAd5 vaccine. Based on these results, a ChAdOx1 F1-V fusion vaccine has progressed to phase I human clinical trials.

INTRODUCTION

Plague is a severe zoonotic infectious disease caused by *Yersinia pestis* and remains endemic in several regions of Africa, Asia, and the Americas,[1] and plague has been known as a biothreat agent for many decades.[2] It is now considered a re-emerging pathogen as a zoonotic disease with large reservoirs in Africa, Asia, and the Americas.[3] Its endemicity worldwide results in sporadic infections and recent outbreaks on different continents,[4–7] with surprisingly large epidemics in Madagascar in 2017–2018.[8] Moreover, the COVID-19 pandemic lowered the capacity of the country to face plague outbreaks.[9] There are three forms of human plague: bubonic, septicemic, and pneumonic.[1] Pneumonic plague results in rapid human-to-human transmission and leads to near 100% mortality unless antibiotic treatment is started within 24 h of symptoms onset.[1] Antibiotic therapy is currently the only method for treating plague, and there are reports of streptomycin-resistant strains emerging.[10]

Previous vaccine development efforts against plague focused on the use of live attenuated and subunit protein vaccines. A live attenuated vaccine has been used since 1934 but is associated with safety and reactogenicity concerns, and protection does not persist after 12 months post-vaccination, making it unsuitable for low-income countries.[11] Subunit proteins administered with an adjuvant induce protection in animal models.[12] These vaccines have demonstrated the protective capacity of fraction 1 antigen (F1, encoded by the operon *caf1*) and V, also named LcrV, a virulence factor involved in the secretion system of the bacteria and carried by the low calcium response (lcr) plasmid. Both antigens induce protection against plague in animal models, generate protective antibodies during natural infection, and have been investigated in clinical trials.[13,14] The immune response to subunit vaccines is predominantly humoral. However, T-cell responses have been associated with increased protection in animal models.[15]

Received 25 June 2025; accepted 17 February 2026;
https://doi.org/10.1016/j.ymthe.2026.02.036.

[7]Present address: Moderna Inc., Cambridge, MA, USA.

[8]These authors contributed equally

Correspondence: Young Chan Kim, Oxford Vaccine Group, Department of Paediatrics, University of Oxford and the NIHR Oxford Biomedical Research Centre, CCVTM, Churchill Lane, OX37LE Oxford, UK.
E-mail: young.kim@paediatrics.ox.ac.uk

 © 2026 The Author(s).
Published by Elsevier Inc. on behalf of The American Society of Gene and Cell Therapy.
This is an open access article under the CC BY license (http://creativecommons.org/licenses/by/4.0/).

The adenoviral-based vaccine technology is suitable when cellular immune responses along with antibody responses are required for protection.[16,17] Moreover, this approach is suited to outbreak situations in low-income countries because of its low cost, as shown for Ebola[18] and SARS-CoV-2.[19,20] While expression of some bacterial antigens in eukaryotic cells can result in aberrant conformation or post-transcriptional modifications,[21] the *Y. pestis* F1 and V antigens can be expressed successfully in poxviral,[22] adenoviral, and mRNA vaccines.[23–26] Here, we constructed a series of adenoviral-vectored vaccines encoding F1, V, or both. Immunogenicity and protection were evaluated, using a murine lethal aerosolized challenge model.

RESULTS

All vaccine candidates are immunogenic in mice

We previously observed that modification of the bacterial signal sequence included in the transgene can impact the resulting immunogenicity.[21,27,28] In particular the use of a dual-signal sequence including both the mammalian tissue plasminogen activator (tPA) signal sequence and the bacterial signal peptide appeared beneficial.[27] Therefore, to test the effect of the signal sequence on antigen expression and immunogenicity, we constructed four monovalent adenoviral-vectored vaccines expressing a single antigen (F1 or V) (Table S1; Figure S1). Three versions of an expression cassette expressing F1 were designed that differed in the bacterial signal sequence used after the tPA signal: either full-length F1 bacterial signal sequence (FL), thus containing two signal sequences; no F1 signal sequence (T, truncated), thus containing only the tPA signal sequence; or the F1 signal sequence replaced with the group B meningococcal (MenB) fHbp bacterial signal sequence (fHbpSS), previously shown to impact antigen expression[28] (Figure S1). A single design including tPA was generated for V, as it does not contain a signal sequence (Table S1; Figure S1). Antigen expression was assessed 18 h after infection of HeLa cells. Preserving the wild-type F1 signal sequence in the resulting adenoviral vector led to the highest level of F1 antigen expression in HeLa cells (4.3% of cells with fluorescence >250) compared with the absence of signal sequence (T) (0.31%) and the MenB fHbp signal sequence (0.53%, Figure S2).

In a first *in vivo* experiment, the vaccine constructs expressing the F1 or V antigen alone induced antigen-specific serum IgG antibody responses in mice, while naive mice did not (Figure 1). Groups of six mice were immunized with one dose of the monovalent adenoviral-vectored vaccines (Figure 1A). All vaccine vectors induced higher F1 and/or V antibody responses against the relevant antigen, whether alone or mixed, as compared with the control group (naive) (Figures 1B and 1C). There was no evidence of a statistically significant effect of the bacterial signal sequences on the antibody response to F1 at week 6 after injection (Figure 1B), suggesting that the difference of antigen expression levels detected by flow cytometry in cell lines cannot be used as predictor of immunogenicity. In an independent experiment, a positive control group was immunized with 10 μg each of F1 and V recombinant proteins, mixed and adjuvanted with aluminum adjuvant. This subunit vaccine was previously shown to induce protective responses in mice after a two-dose schedule (12, 14). Results showed that a single dose of the adenoviral vectors induced antibody titers comparable with two doses of the adjuvanted protein vaccine (Figures 1D and 1E). We explored whether mixing the F1 and V-expressing vectors would induce immune interferences (Figures 1B and 1C). Results showed that mixing two vectors encoding for F1 and V did not decrease the V-specific antibody responses compared with immunization with a single adenoviral vector expressing V antigen only (Figure 1C). Lower F1-specific antibody titers were observed in the group immunized with a mix using HuAd5 F1 T + HuAd5 V (Figure 1B), while the IgG titers to monovalent F1 FL and F1 T were not statistically significant (Figure 1B).

As vaccine manufacturing would be simpler with a single component, we assessed the impact of combining the two antigens, F1 and V, into a single adenoviral vector (fusion constructs) (Figures 1D and 1E). The vectors were designed to encode a fusion F1-V protein, with the two antigens separated by a flexible linker. As for the monovalent F1 constructs, three versions of the F1 expression cassette were used, differing in the bacterial signal sequence placed downstream of the tPA signal: FL, T, or fHbpSS. Two linkers (L1 and L2) were investigated (Table S1). In total, five fusion F1-V immunogens were produced and inserted into adenoviral vectors (HuAd5): (1) F1 T (L1) V, (2) F1 T (L2) V, (3) F1 FL (L1) V, (4) F1 FL (L2) V, and (5) fHbpSS F1 (L1) (summarized by Table S1). Prior to immunogenicity experiments, antigen expression from the fusion constructs was confirmed on the surface of HeLa cells (Figure S2).

Mice were immunized with a single dose of adenoviral-vectored vaccine, and responses were compared with the monovalent vectors alone or mixed, as well as with two doses of the adjuvanted F1+V protein vaccine. At week 6, F1-specific antibody responses elicited by the five HuAd5 fusion F1-V vaccines were broadly similar to those induced by the monovalent HuAd5 F1 constructs (FL, fHbpSS, T) (Figure 1D), except for the fusion construct incorporating the fHbp signal sequence (fHbpSS F1 [L1]) which induced a lower F1-specific antibody responses than the F1+V protein control (Figure 1D, $p = 0.0167$). V-specific antibody responses were lower for three fusion constructs encoding F1 FL (L1) V, F1 FL (L2) V, and fHbpSS F1 (L1) V than with HuAd5 V alone (Figure 1E, $p = 0.0042$, 0.0021, and 0.0439, respectively), whereas the two constructs encoding F1 T (L1) V and F1 T (L2) V did not differ significantly from HuAd5 V alone. There were no statistically significant differences between any of the fusion constructs and the F1+V protein control. Overall, these data show that single-dose adenoviral-vectored fusion vaccines can elicit strong antibody responses to both F1 and V, with only a limited reduction in titers when both antigens are combined into a single construct.

A single-dose vaccine induces early and persistent antibody response

We investigated the persistence of antibody response following a single immunization with single adenoviral vectors encoding the fusion constructs, compared with two injections of adjuvanted recombinant F1+V protein, up to week 34 (Figure 2A). As multiple HuAd5 F1

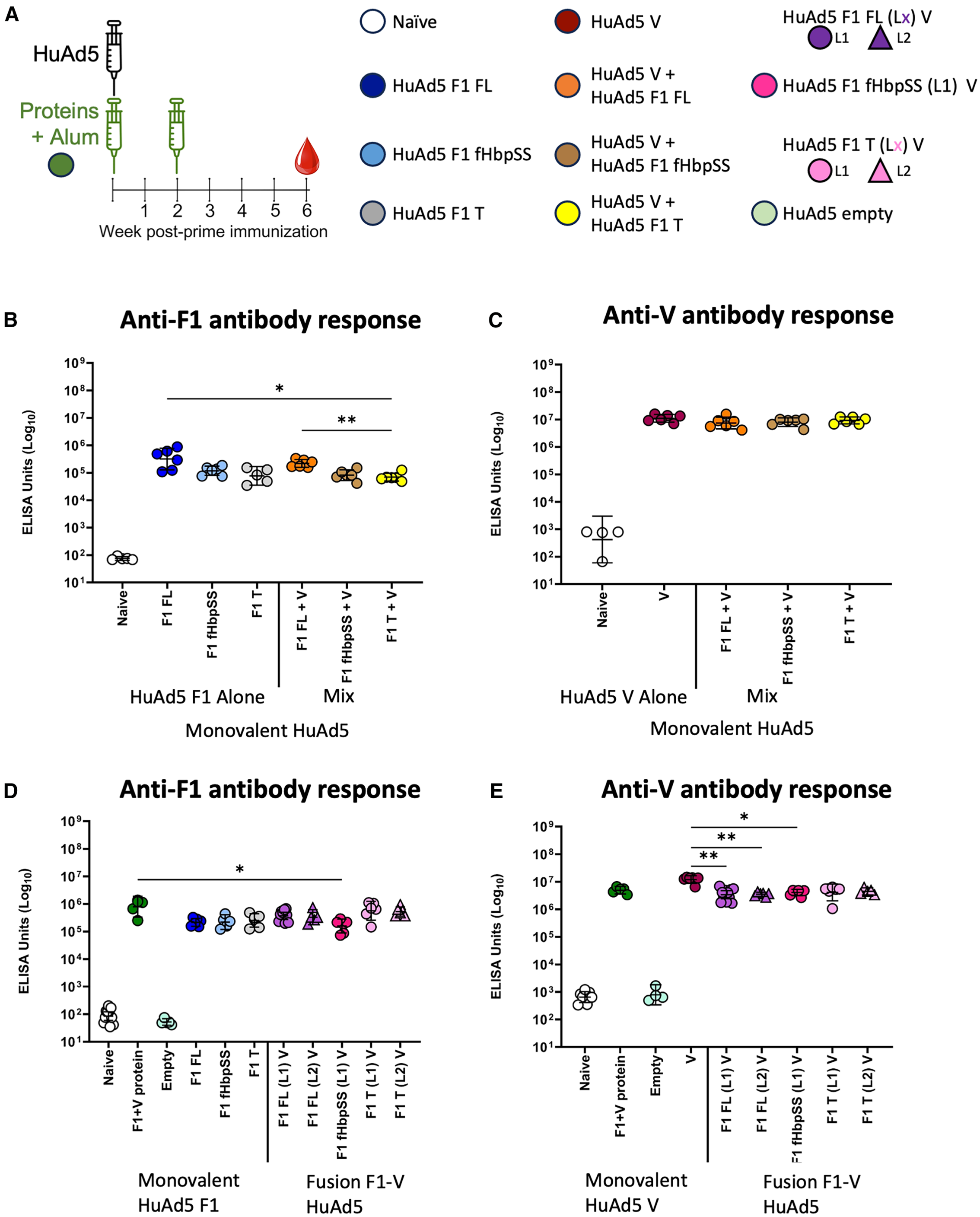

Figure 1. The vaccines containing F1 and/or V, alone or mixed, elicit F1- and V-specific serum antibody titers in mice after a single dose
Groups of NIH Swiss mice (n = 4 to 12) were immunized via intramuscular route at day 0 with either 10^9 infectious units of HuAd5 as indicated in the legend or at day 0 and 14 with 10 μg of each recombinant F1 and V proteins were formulated in aluminum adjuvant (A). The HuAd5 vectors expressed F1 or V alone, or individual vectors mixed, or constructs expressing F1 and V fusion proteins using linkers L1 (circles) or L2 (triangles), as indicated in the legend (A) and X axis of each panel (B–E). Antibody responses were measured at week 6 against F1 (B and D) and V (C and E). Negative controls were mice immunized with an empty adenovirus (pale green) or non-immunized naive mice (white) as indicated. Positive controls consisted of mice immunized with recombinant F1 and V proteins formulated in aluminum adjuvant, administered twice (dark green). Individual data are presented as well as median ±95% confidence intervals for each group. The experiment shown in (A) and (B) was independent of experiments shown in (D) and (E). Data were analyzed by Kruskal-Wallis with Dunns multiple comparison test (*p < 0.05 and **p < 0.01).

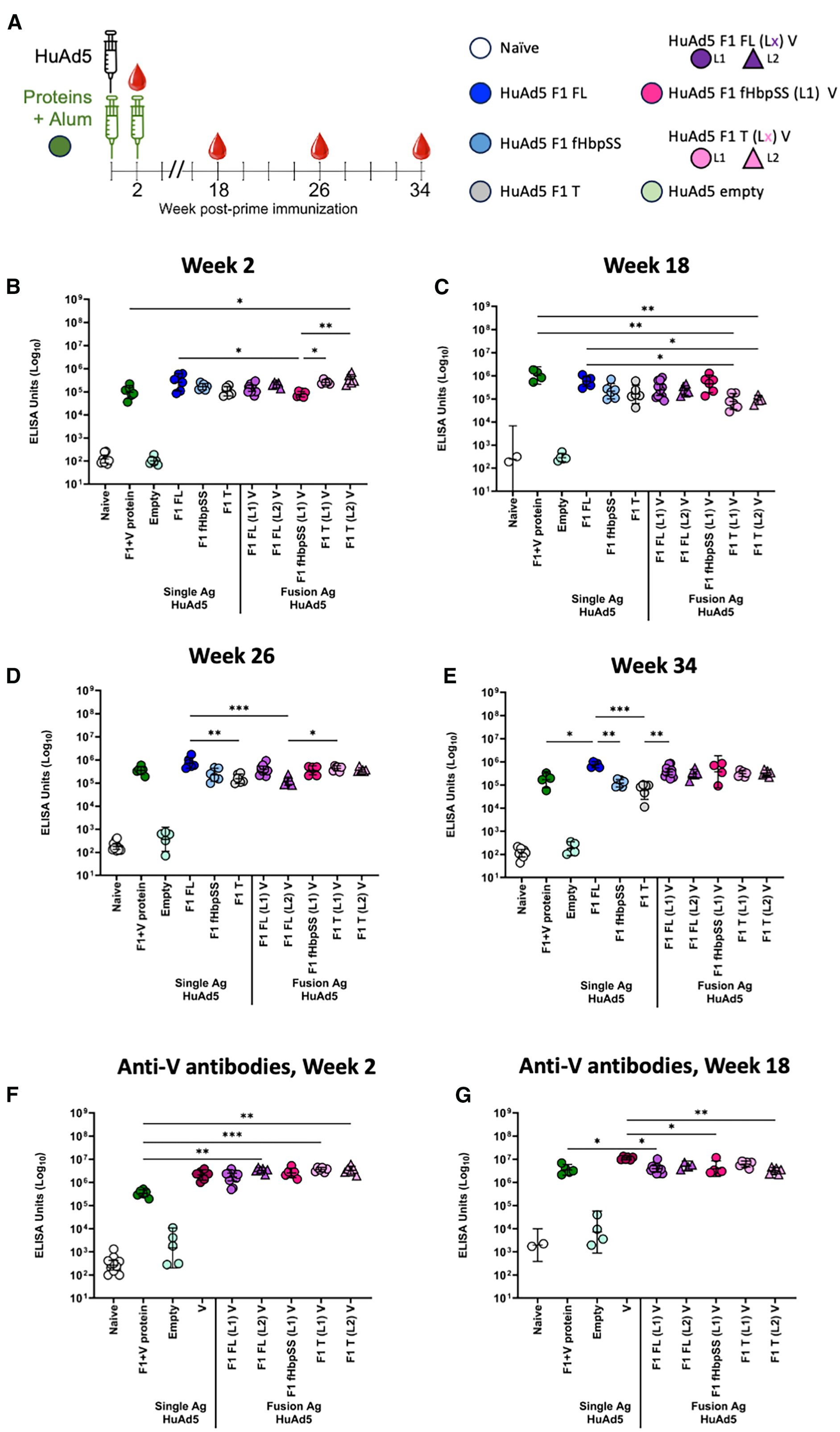

(legend on next page)

designs (FL, fHbpSS, and T) were evaluated in both monovalent and fusion formats to assess the impact of the F1 signal sequence on expression and immunogenicity, anti-F1 ELISAs were performed longitudinally at weeks 2, 18, 26, and 34. In contrast, V was tested as a single monovalent design (tPA-V) without signal sequence variants; therefore, anti-V ELISAs were performed at weeks 2 and 18 only.

F1-specific antibody responses were detected in all F1 and V vaccinated groups as soon as week 2 (Figure 2B). The fusion vector incorporating the fHbp signal sequence induced lower responses than the fusion vectors using either L1 or L2 linker (p = 0.0148 and 0.0015 respectively). This group also had lower antibody response than the monovalent HuAd5 F1 FL vector (p = 0.0438). Both vectors encoding the fusion protein using the F1 full length (FL) signal sequence induced similar antibody levels to the monovalent HuAd5 F1 FL (Figure 2B).

At week 18, there was little decline observed in most groups except with the fusion constructs using the truncated F1 T (no signal sequence) irrespective of the L1 or L2 linker (Figure 2C), as F1 antibody titers became significantly lower than after two doses of recombinant protein (p = 0.0051 and 0.0087, respectively), or after a single dose of the monovalent F1 FL vaccine (p = 0.0135 and 0.0244, respectively). At that time point, the antibody titers were highest in the group immunized with HuAd5 F1 FL and lowest in the HuAd5 F1 T and F1 FL (L2) V (Figure 2D). At week 34, the antibody titers decreased in the groups immunized with the monovalent HuAd5 F1 T and F1 fHbpSS vectors, and with the recombinant proteins, while the titers in mice immunized with HuAd5 F1 FL and the fusion vaccine HuAd5 F1 FL (L1) V remained high (Figure 2E). These results show that the anti-F1 antibody titers remained high after two doses of the adjuvanted protein mix or a single dose of adenoviral vaccine.

The anti-V antibody responses were also measured at weeks 2 and 18 (Figures 2F and 2G). At week 2, a single immunization with vectors encoding the fusion of F1-V induced anti-V responses that were similar or higher than those elicited after the first protein immunization (Figure 2F). Following the second protein immunization, this difference was abrogated, and the monovalent HuAd5 expressing V only induced higher antibody titers, followed by the fusion constructs incorporating the full-length F1 variant (Figure 2G). Altogether, these results demonstrate that a single HuAd5 dose induces persistent antibody response comparable to two doses of adjuvanted recombinant F1+V proteins. Although differences were observed between constructs, the most consistently high-performing vaccines were monovalent F1 FL and V vaccines and the fusion construct F1 FL (L1) V.

Mucosal delivery of the adenoviral vaccines induces systemic antibody responses

Adenoviruses can be delivered by mucosal routes, owing to their capacity to infect different cell types, so we assessed the systemic immunogenicity elicited by intranasal and sublingual administration of a selection of the vaccine candidates (Figure 3). Our results show that the intranasal route induced systemic antibody responses in all mice (Figures 3B and 3C). The sublingual route induced more variable serum responses than the other two routes: some mice did not elicit any antibody response to F1 (Figure 3B), and lower V-specific responses than the intramuscular route were detected (Figure 3C).

A single-dose vaccine protects against a virulent aerosol challenge

To assess the protective efficacy of the vaccine candidates, an *in vivo* aerosol challenge experiment was performed using NIH Swiss mice (Figure 4A). The experiment included the three vaccine candidates encoding the two antigens F1 and V fused, differing in the signal sequence used (FL, fHbpSS, and T). Adenoviral-vectored vaccines encoding each antigen alone or combined in a mixture (the two monovalent vectors mixed in the syringe) were used as comparators. The positive control consisted of mice immunized with the recombinant proteins in alum adjuvant, administered at days 0 and 14. The negative control group consisted of naive mice. Moreover, a group immunized by the intranasal route was added, owing to the systemic immunogenicity observed with this route. Due to constraint in experiment size, only the fusion vector containing the fHbp signal sequence was selected for intranasal delivery (Figure 4A). The selection of HuAd5 F1 fHbpSS IN group was based on the serum antibody responses induced by the HuAd5 plague IN groups (Figures 3B and 3C). The serum anti-V antibody response induced by HuAd5 F1 fHbpSS IN group was higher than that of F1 FL (L1) V, while the anti-F1 antibody responses by both groups were comparable. Antibody responses were assessed 1 week prior to challenge and demonstrated that all vaccines were immunogenic and induced similar anti-F1 and/or anti-V antibody titers, with the exception of the intranasal delivery, which induced lower serum IgG responses than the other vaccines, in particular with regard to F1-specific antibodies (Figures 4B and 4C).

After the aerosol challenge with *Y. pestis* CO92, 80% of untreated mice were reached the humane endpoint and were euthanized or died between observations (Figure 4D). All control mice displayed

Figure 2. A single dose of adenovirus vaccines induces a persistent serum antibody response in mice

Eleven groups of NIH Swiss mice (n = 6 to 12) were immunized with the vaccine via intramuscular route as described in Figures 1A–1E, with the vaccines indicated in the legend (A) and X axis of each panel. A single dose of 10^9 infectious unit of adenoviral vaccines was injected at day 0, while 10 μg of each recombinant F1 and V proteins formulated in aluminum adjuvant was injected at day 0 and 14 (A). The kinetics of the F1-specific serum antibody response is shown at weeks 2, 18, 26, and 34 (B–E), and the V-specific serum antibody response was assessed at weeks 2 and 18 (F and G). Individual data are presented as well as median ±95% confidence intervals for each group. Not all sera could be collected or assessed at all time points in this longitudinal study, so the number of sera tested per time point and for each ELISA is provided in Tables S2 and S3. Data were analyzed by Kruskal-Wallis with Dunns multiple comparison test (*p < 0.05 and **p < 0.01).

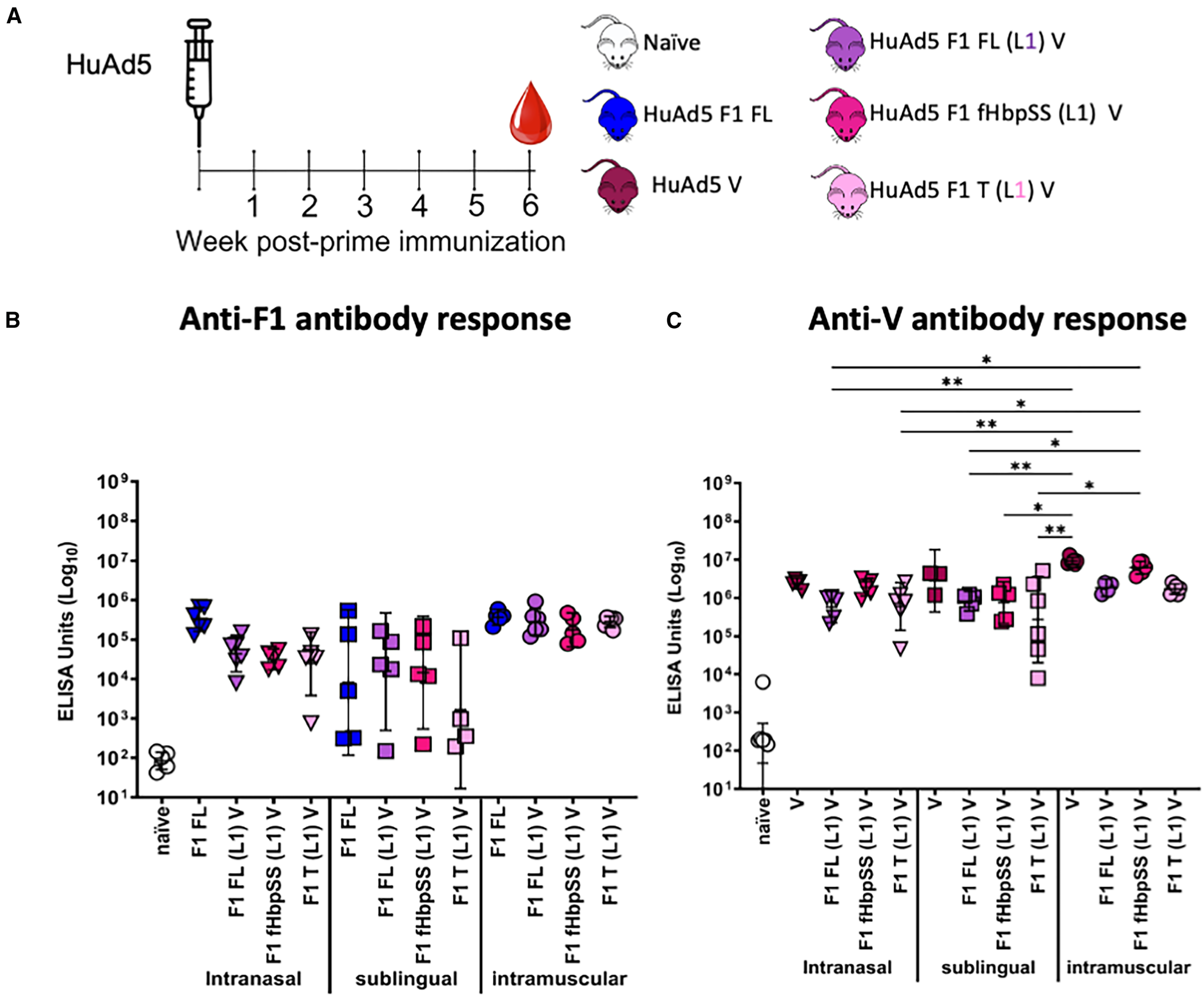

Figure 3. Serum antibody responses are induced by the HuAd5 plague vaccines administered by the mucosal routes

Sixteen groups of NIH Swiss mice ($n = 6$) were included in the study. One group remained naive (white circles), and the fifteen other groups were immunized with a single dose (10^9 infectious unit) of either one of the five vaccines described in (A) and indicated in the X axis of each panel (B and C), by either the intranasal (triangles), sublingual (squares), or intramuscular route (circles). Antibody responses were measured at week 6 against F1 (B) and V (C). Individual data are presented with median ±95% confidence intervals for each group. Data were analyzed by Kruskal-Wallis with Dunns multiple comparison test (*$p < 0.05$ and **$p < 0.01$).

clinical signs of illness (Figure 4E), and of those untreated controls that were euthanized or died between observations, death occurred within 4 days of the infectious exposure (Figure 4F). All mice in the three groups immunized with either one of the fusion vaccines by the intramuscular route and mice immunized with two doses of adjuvanted recombinant proteins survived for three weeks (100% survival, Figures 4D and 4F). Importantly, in addition to 100% protection from mortality, all mice immunized with a single dose of HuAd5 F1 FL (L1) V also remained healthy (no clinical signs of disease) throughout the follow-up (Figure 4E). In contrast, although protected from mortality, 60% of mice immunized with a single dose of HuAd5 F1 T (L1) V or two doses of the adjuvanted recombinant proteins displayed clinical signs of infection. All surviving mice in the other groups displayed clinical signs of illness after infection (Figure 4E). Mice immunized with HuAd5 V alone or HuAd5 F1 mixed with HuAd5 V were partially protected from mortality (90% survival in each group, all with clinical signs of illness), suggesting that the immune response to V has a greater protective impact than the response to F1 in mice. Unlike the naive mice, some deaths (euthanasia at humane endpoint or death between observations) that occurred in mice immunized with the F1 encoding adenoviral vaccines were spread out over an extended period, with the last death occurring as late as day 18 after infection (Figure 4D).

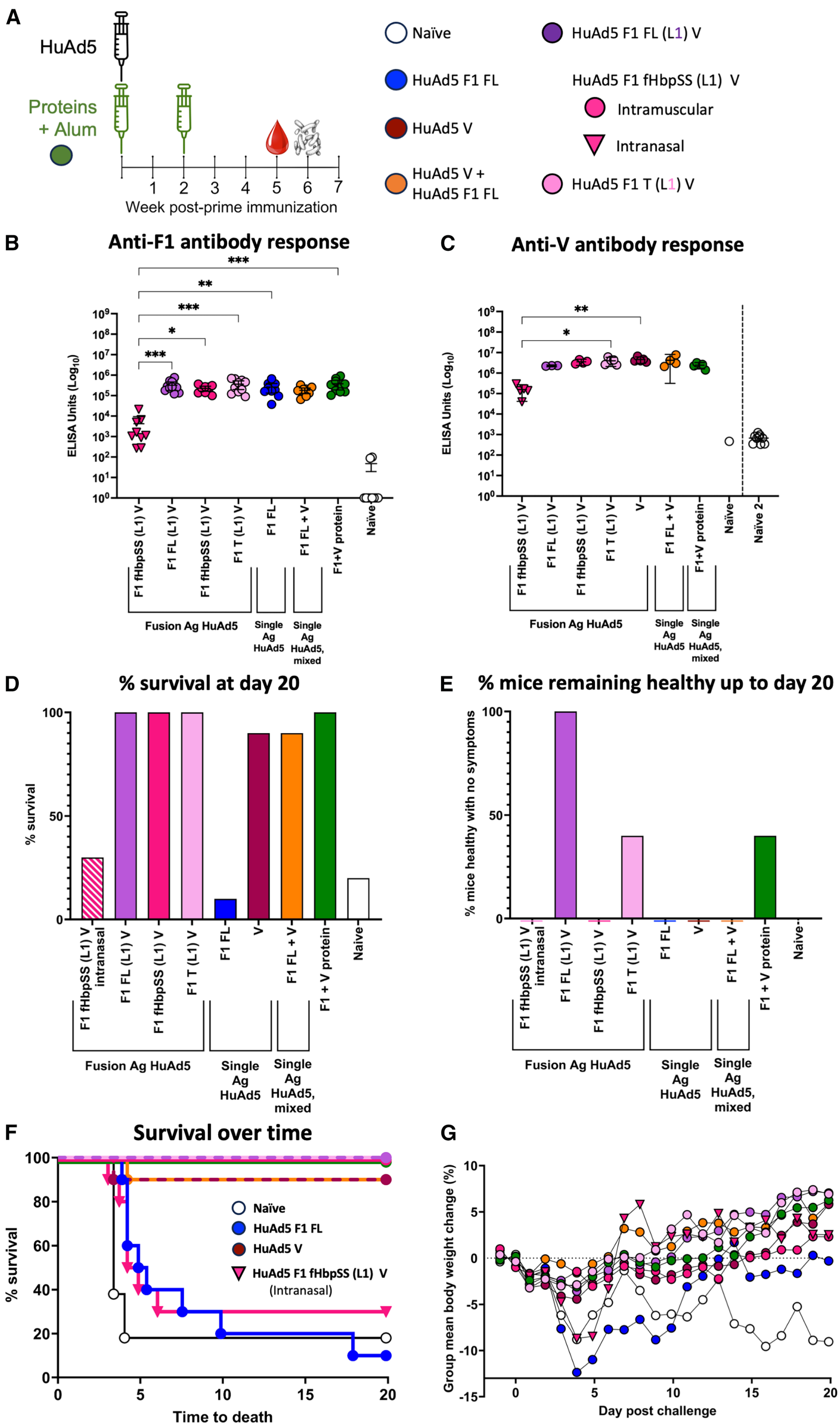

(legend on next page)

Mice immunized with the fusion HuAd5 F1 fHbpSS (L1) V by intranasal route were not protected following aerosol infection (30% survival, Figures 4D and 4F), and all survivors displayed clinical signs of illness (Figure 4E). All of the deaths in the group immunized intranasally with HuAd5 F1 fHbpSS (L1) V occurred within the first 6 days following aerosol exposure.

Most deaths were preceded by a short duration of illness characterized by a combination of clinical signs of infection, and some clinical signs appeared and then resolved in survivors (Figure S3). In the naive mice, most death were preceded by a set of symptoms whose score rapidly approached a total score of 9 within 4 days. In the four immunized groups that were 100% protected from mortality (immunized with either of the three fusion constructs or the two doses of adjuvanted recombinant protein by intramuscular route), one group, immunized with HuAd5 F1 FL (L1) V, stood out in not having any recorded clinical signs of infection at all in the 3 weeks following *Y. pestis* CO92 aerosol challenge (Figures 4E and S3).

Group mean body weight data appeared to reflect the outcome of the other clinical signs of infection (Figure 4G). In general, a decrease of at least 10% in body weight preceded euthanasia but was not an accurate predictor of survival or death. The control untreated challenged mice all showed weight loss before death (where it occurred) to varying degrees before euthanasia, and the two survivors failed to regain weight to consistently exceed pre-challenge levels. This may suggest that the mice may not have survived if the time course of the experiment had been extended but no other signs were detected. The groups with 100% survival generally contained mice that gained weight after infection, but exceptions occurred in all groups apart from mice immunized with HuAd5 F1 FL (L1) V, in which all mice met or exceeded pre-infection body weight by day 20 post-infection.

In summary, a single dose of any of the three fusion antigen adenoviral vaccines resulted in 100% survival from lethal *Y. pestis* aerosol infection, and, remarkably, none of the mice immunized with HuAd5 F1 FL L1 V displayed any clinical sign of infection. Mice immunized with the vector using the fHbp signal sequence also survived the infection (100% survival), but all displayed clinical signs of infection. This vaccine candidate delivered by the intranasal route induced lower levels of protection (30% survivors). Interestingly, the mix of HuAd5 F1 FL and HuAd5 V elicited a lower level of protection than the fusion vectors (90% survivors against 100% in the group immunized with HuAd5 F1 FL [L1] V and 100% symptomatic versus 0% symptomatic, respectively).

A single-dose vaccine induces T-cell responses

As antibody responses were similar between the groups immunized intramuscularly with the HuAd5 fusion constructs, the HuAd5 expressing F1 and V mixed, and the protein vaccines, we hypothesized that the antigen-specific T-cell responses may have driven the difference in protection. To address this question, groups of mice were immunized intramuscularly with the vaccines used for the challenge study (Figure 5A), and F1- and V-specific T-cell responses were measured in spleens by IFN-γ and IL-5 T-cell ELISPOT. No IL-5 T-cell responses were detected in any of the groups (data not shown). All adenoviral vaccines given intramuscularly induced high T-cell responses against both antigens, while the adjuvanted proteins did not (Figures 5B and 5C). No statistically significant difference was observed between the adenoviral-based vaccines (Figures 5B and 5C), suggesting that the splenic antigen-specific IFN-γ response is not involved in the lower protection observed in the mice that received the mix compared with the fusion constructs.

Transfer to the clinically relevant backbone ChAdOx1

The immunogenicity of the human adenovirus serotype 5 may be reduced in humans due to pre-existing immunity. The chimpanzee adenovirus serotype ChAdOx1[29] was selected for development of a clinically relevant vaccine against plague. The cassette F1 FL (L1) V was inserted into ChAdOx1, resulting in the clinical vaccine candidate ChAdOx1 plague. Antigen expression in human cells was verified for three clones by western blot. The fusion protein was detected both in cells and in the cell supernatant (Figure 6A). The fusion protein remained mostly intact within the cells (main band detected at approximately 50 kDa size, corresponding to 36 kDa for V plus 15.5 kDa for F1). Immunogenicity of both preclinical and clinical batches of ChAdOx1 plague vaccine was confirmed in independent experiments in outbred NIH Swiss mice (Figures 6B–6D). Groups of NIH Swiss mice ($n = 6$) were immunized by intramuscular route with a single dose of 10^8 infectious units of ChAdOx1 plague or HuAd5 plague, or 10 μg of each recombinant F1 and V formulated in alum adjuvant (Figure 6B), and the serum IgG response specific for F1 and V was measured at week 6 (Figures 6C and 6D). Groups of preclinical and clinical batch of ChAdOx1 plague induced serum IgG responses to both F1 and V that were comparable to those

Figure 4. HuAd5 F1 L1 V induces full protection against a virulent plague aerosol challenge in mice

Nine groups of NIH Swiss mice ($n = 10$) were included in the study. One group remained naive (white), and one group was immunized twice by intramuscular route with 10 μg of each recombinant F1 and V protein formulated in aluminum adjuvant at days 0 and 14 (positive control, green). The other groups were immunized with a single dose (10^9 infectious unit) of either one of the HuAd5 vaccines described in (A) and indicated in the X axis of panels (B) and (C). All vaccines were administered by the intramuscular route except for one group that received HuAd5 F1 fHbp SS (L1) V by the intranasal route (A). One mouse died for unrelated reason in this group, reducing the number to nine animals. Antibody responses were measured at week 5, prior to challenge, against F1 for all mice (B) and against V for a subset of mice (C); since only one naive mouse for that group was assessed for logistical reasons (Naive), a second group of naive mice is represented (Naive 2). Individual data are presented with median ±95% confidence intervals for each group, except for the group with only one datapoint (Naive in the anti-V ELISA). Data were analyzed by Kruskal-Wallis with Dunns multiple comparison test (*$p < 0.05$, **$p < 0.01$, ***$p < 0.001$). Mice were challenged by aersol at week 6 and followed for 20 days post challenge for clinical signs of illness. Panels show survival at the final time point (day 20) (D), the percentage of mice remaining healthy until the final follow-up day (day 20) (E), survival over time after challenge (F), and the mean percentage change in body weight over time among surviving mice in each group (G).

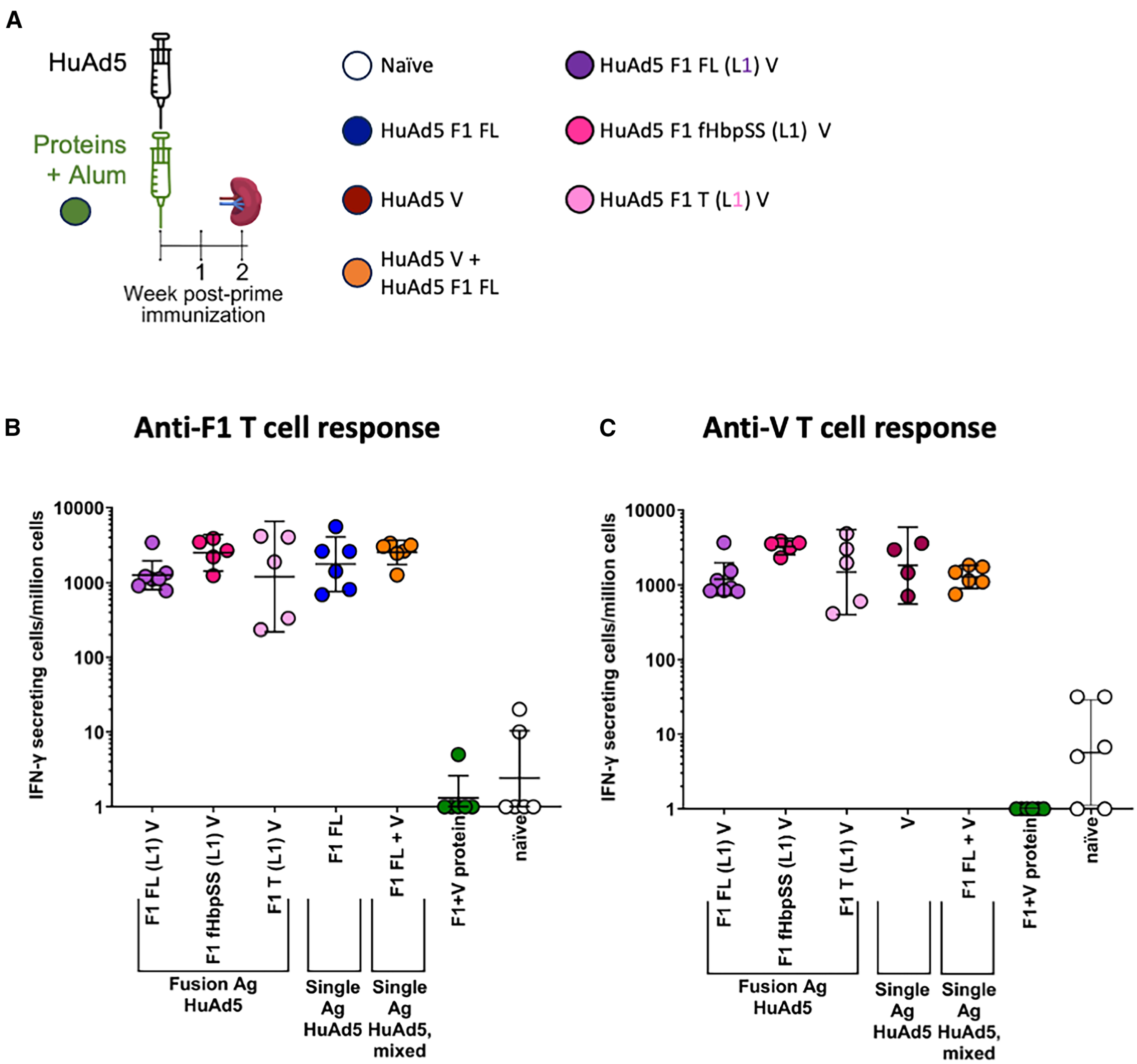

Figure 5. IFN-γ secreting T-cell responses as measured by ELISPOT are not associated with the difference in protection capacity elicited by the vectored vaccines

Groups of NIH Swiss mice (n = 5 to 7) were immunized by intramuscular route with as indicated in the legend (A) with a single dose of 10^9 infectious units of adenovirus or 10 μg of each recombinant F1 and V proteins formulated in aluminum adjuvant, and T-cell responses were measured in splenocytes against a peptide pool covering F1 (B) or V (C) at week 2. Shown are individual responses with geometric mean and 95% confidence intervals for each group. Statistical analysis was performed to compare the adenovirus vaccinated groups, and no statistical differences were observed between these groups.

observed with HuAd5 plague (Figures 6C and 6D). Next, immunogenicity was also confirmed in an independent experiment in outbred CD-1 mice (Figures 6E–6I). Groups of mice were administered a single dose of either HuAd5 or ChAdOx1 plague at the doses ranging from 10^5 to 10^8 infectious units. The serum IgG and spleen T-cell responses to F1 (Figures 6E and 6H) and V (Figures 6G and 6I), respectively, were measured at week 6. ChAdOx1 plague vaccine was shown to induce dose-dependent response for both anti-F1 and V antibody responses and anti-F1 and anti-V T-cell responses (Figures 6E–6I). Similarly, dose-dependent response of ChAdOx1 plague vaccine was also confirmed in NIH Swiss mice (Figures S4A–S4C). Immunogenicity was also confirmed when ChAdOx1 plague was delivered by the subcutaneous route (Figures S4D and S4E). Using this route, a higher dose of ChAdOx1 plague was necessary to induce 100% response (5×10^7 infectious units) compared with the intramuscular route (5×10^5 infectious units).

Lastly, an exploratory experiment was performed to compare T-cell immunogenicity induced by ChAdOx1 plague when administered by the intramuscular (IM) versus the sublingual (SL) route (Figure 7). For this route comparison, we assessed F1-specific IFNγ T-cell responses as a representative antigen readout. The IM route induced

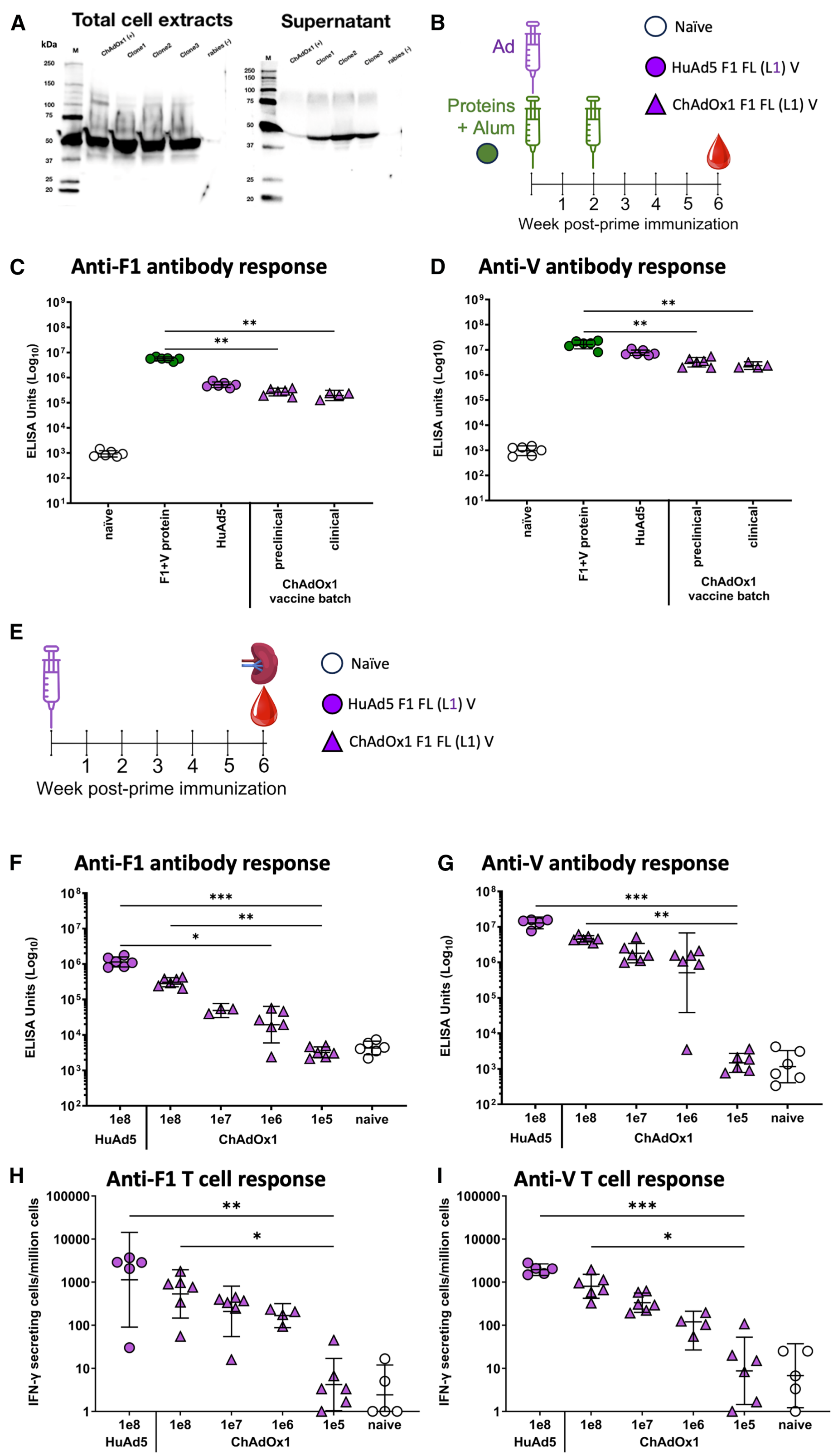

(legend on next page)

the strongest antigen-specific IFNγ T-cell response in the spleen (Figures 7B and 7C), whereas lung T-cell responses were comparable when ChAdOx1 or HuAd5 were administered by either the IM or SL route (Figures 7D and 7E). In all cases, F1-specific T-cell responses were higher following adenoviral vector immunization than after the protein formulation. LcrV-specific T-cell responses were measured in the spleen following ChAdOx1 plague IM immunization and are shown in Figure 6I, where they were of similar magnitude to the F1-specific responses (Figure 6H); however, LcrV-specific responses in spleen and lungs were not assessed in this exploratory route-comparison experiment.

Altogether, these results indicate that a clinically relevant ChAdOx1 vaccine vector encoding the two plague antigens F1 and V as a fusion protein is immunogenic *in vivo* in mice and induces similar responses to the HuAd5 counterpart.

A single dose of ChAdOx1 vaccine provides similar protection to HuAd5 vaccine in a virulent aerosol challenge

An *in vivo* aerosol challenge experiment using NIH Swiss mice was performed to assess the protective efficacy of the ChAdOx1 vaccine (Figure 8). The experiment included the ChAdOx1 plague vaccine candidate (F1 FL [L1] V) and the HuAd5 plague (F1 FL [L1] V), which provided full protection following a single dose of 1×10^9 infectious unit after the aerosolized *Y. pestis* CO92 challenge study with an estimated dose of 6.5 MLD50 (3.25E+04 CFU) (Figure 4). After a higher aerosol challenge dose of 9.2 MLD50 (4.6E+04 CFU), all of the naive mice displayed clinical signs of illness and reached the humane endpoint and were euthanized or died by day 5 (Figures 8A and 8B). Nine out of ten mice in the two groups immunized with either 5×10^8 infectious unit of ChAdOx1 or HuAd5 vaccines by the intramuscular route, survived for 2 weeks (90% survival, Figures 8A–8D). Importantly, these mice remained healthy with clinical score of <2 with stable group mean body mass percentage change and mean group temperature change throughout the follow-up (Figure 8B).

In summary, a single dose of both adenoviral vaccines (ChAdOx1 or HuAd5) encoding the same antigen resulted in 90% survival from lethal *Y. pestis* aerosol infection in this study despite higher estimated challenge dose of 9.2 MLD50 of aerosolized *Y. pestis* CO92 compared with the first challenge study using a challenge dose of 6.5 MLD50 (Figure 4), and all surviving ChAdOx1-immunized mice remained healthy with minimal clinical sign of infection similar to HuAd5-immunized mice. The vaccine candidate ChAdOx1 plague has progressed to clinical development.

DISCUSSION

Several adenoviral vectors encoding the protective *Y. pestis* antigens F1, V, or both were constructed and evaluated. All vectors and antigen combinations induced measurable antibody responses in mice. Notably, a single dose of a vector expressing a fusion of F1 and V conferred complete protection mice against lethal challenge with aerosolized *Y. pestis*. The transgene was subsequently inserted into the clinically relevant replication-deficient adenovirus backbone (ChAdOx1), which has been widely used in the Oxford/AstraZeneca SARS-CoV-2 vaccine, and this construct induced protective efficacy comparable to the HuAd5 candidate in a second challenge study using a higher aerosol challenge dose.

The gene encoding for the F1 antigen was inserted as either full length with its bacterial signal sequence followed by the protein gene or without its bacterial signal sequence. In both cases, a tPA signal sequence was added to direct the antigen to the secretion pathway. As a result, two signal sequences were encoded in the full-length constructs, an original design compared with other adenoviral vaccines expressing bacterial antigens.[30–32] These modifications did not alter antigen-specific immune responses for vectors expressing F1 alone or the F1-V fusion. This contrasts with prior observations using the meningococcal antigen fHbp, where inclusion of dual-signal sequence (tPA followed by the fHbp signal sequence) induced higher antibody responses, possibly through improved processing, presentation, or native folding.[27,28] However the precise mechanism underlying the effects of dual-signal sequences remain unclear,[33] and the lack of effect with F1 suggests that the impact of using two signal sequences may be antigen specific. Moreover, the meningococcal fHbp signal sequence also did not increase the immune response and decreased the protective capacity. This suggests that use of the natural signal sequence is favorable, as observed for SARS-CoV-2 S antigen.[33]

Antibody responses persisted after a single injection of the adenoviral vectors in mice, in agreement with previous findings using another bacterial antigen.[27] Persistence of antibody responses after a single adenoviral vaccine injection in humans has been observed after a single dose of Ebola vaccine in children.[34] However, better responses and longer persistence against SARS-CoV-2 were observed after a

Figure 6. Antigen expression and immunogenicity of the clinically relevant ChAdOx1 plague vaccine candidate

Antigen expression in infected HeLa cells (A). Cells were infected with a preclinical-grade ChAdOx1 plague (positive control) or three clones of clinically generated ChAdOx1 plague. Cell pellets and supernatants were assessed for expression of the fusion antigen by western blot using an anti-V monoclonal antibody. Infection of cells with a ChAdOx1 vector expressing a rabies antigen was used as negative control. The molecular weight of V is 36 kDa, the molecular weight of F1 is 15.5, and the expected molecular weight of the fusion protein in the fusion construct is 51.5 kDa. ChAdOx1 plague is immunogenic in inbred (B–D) and outbred mice (E–I). Groups of NIH Swiss mice ($n = 6$) were immunized by intramuscular route with a single dose of 10^8 infectious units of ChAdOx1 plague (purple triangles, two different production batches) or HuAd5 plague (purple circles), or 10 μg of each recombinant F1 and V formulated in alum adjuvant (green) (B). The serum IgG response specific for F1 (C) and V (D) was measured at week 6. Immunogenicity was also confirmed in an independent experiment in outbred CD-1 mice (E–I). Groups of mice were administered a single dose of either HuAd5 (purple circles) or ChAdOx1 (purple triangles) plague (E) at the doses indicated in the X axis of each panel (10^5 to 10^8 infectious units). The serum IgG and spleen T-cell responses to F1 (E and H) and V (G and I), respectively, were measured at week 6. Shown are individual responses with geometric mean and 95% confidence intervals for each group. Data were analyzed by Kruskal-Wallis with Dunns multiple comparison test (*$p < 0.05$, **$p < 0.01$, ***$p < 0.001$).

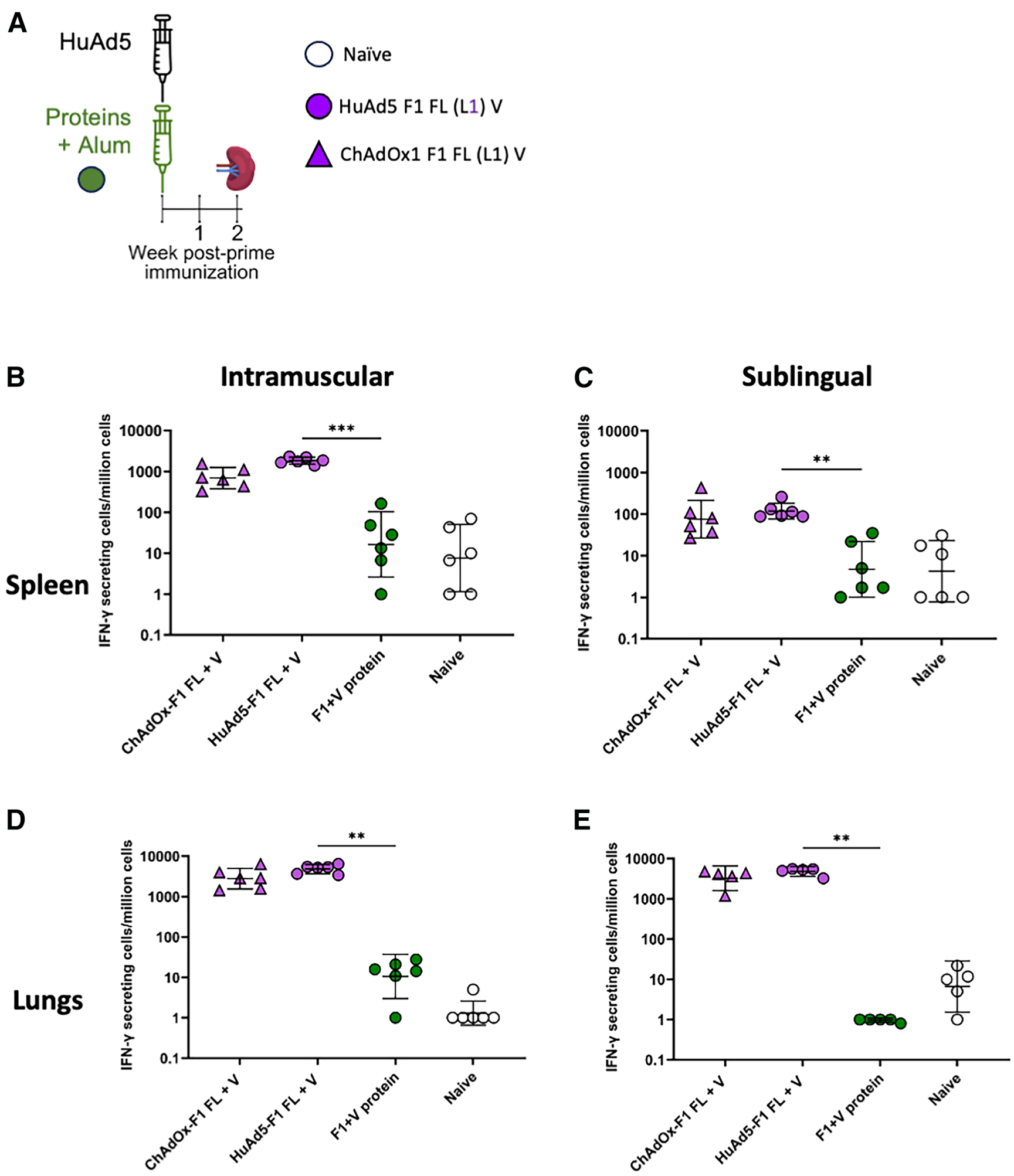

Figure 7. ChAdOx1 plague induces T-cell responses by both the intramuscular and sublingual routes

A single dose of either ChAdOx1 plague (purple triangles) or HuAd5 plague (purple circles), or 10 μg of each recombinant F1 and V formulated in alum adjuvant (green), was administered to groups of NIH Swiss mice (n = 6) (A). The F1-specific T-cell responses were measured at week 2 in spleens (B and C) and lungs (D and E), after intramuscular (B and D) or sublingual (C and E) immunization. Shown are individual responses with geometric mean and 95% confidence intervals for each group. Data were analyzed by Kruskal-Wallis with Dunns multiple comparison test (**$p < 0.01$ and ***$p < 0.001$).

second dose of ChAdOx1 nCoV-19,[35] suggesting that a second dose of the adenovirus-based vaccine may be required in humans.

The survival outcome of each group following challenge was critically affected by pre-challenge vaccination. Every mouse immunized with the fusion constructs or with two doses of the recombinant proteins survived after exposure to an estimated dose of 3.25 + 04 CFU (6.5 MLD50) of aerosolized *Y. pestis*. Mice immunized with HuAd5 V or a mix of HuAd5 expressing F1 and V separately also survived well (80% survival in each). In contrast, no protective effect was evident following dosing with HuAd5 F1 alone or with the adenoviral vaccine given intranasally. Although body weight data reflected the other clinical signs of infection, it was not reliable as a predictor of outcome in terms of survival and cannot be used as a trigger for euthanasia on its own. The lack of any clinical signs of infection or enduring weight loss in mice immunized with a single dose of HuAd5 F1 FL L1 V was particularly notable. The intensive monitoring (day and night for 3 weeks) enabled to differentiate the four groups that demonstrated 100% survival and assisted in the selection of an optimal vaccination formulation.

A limitation of this study is that the bacterial loads in the lung and other organs as well as histopathological analyses were not performed due to logistical and biosafety constraints associated with tissue processing in the aerosol challenge model. As a result, efficacy was evaluated using survival and clinical scores, and we cannot conclude whether vaccination induced sterilizing immunity (complete bacterial clearance) or quantify vaccine-associated reductions in organ pathology. Future studies incorporating quantitative bacteriology and histopathology will be needed to address these important endpoints.

Our findings indicate that optimal protection in this model results from combined anti-F1 and anti-V immune responses, consistent with previous results with recombinant F1 and V,[36] poxviral,[37,38] and adenoviral-based vaccines.[26] A higher protective capacity was also observed with a recombinant V antigen over F1 in guinea pigs.[39] With the poxviral vaccines, fusion of the two antigens elicited similar protection as the mix of individually expressing vectors, while here, the fusion of F1 and V induced better protection than the mix of HuAd5 individually expressing F1 and V.[22] It is possible that mixing two adenoviruses negatively impacts the cell infection rates, while the doses used with the poxviruses avoided this issue. F1 is known to autoassemble using hydrogen bonds and hydrophobic interactions[40]; the multimeric form is more protective.[36] The fusion construct developed here could be responsible for a better presentation of both antigens,[36] as suggested by a recombinant fusion protein-based vaccine.[41]

Despite these findings, antibody titers and T-cell responses did not clearly distinguish the most protective vaccine formulations from monovalent, less protective vaccines. The IgG antibody titers to F1 and V correlate with protection in several animal models.[42] Anti-F1 and V antibody or serum transfer from animals or humans immunized with these two antigens also induces protection.[43,44] However

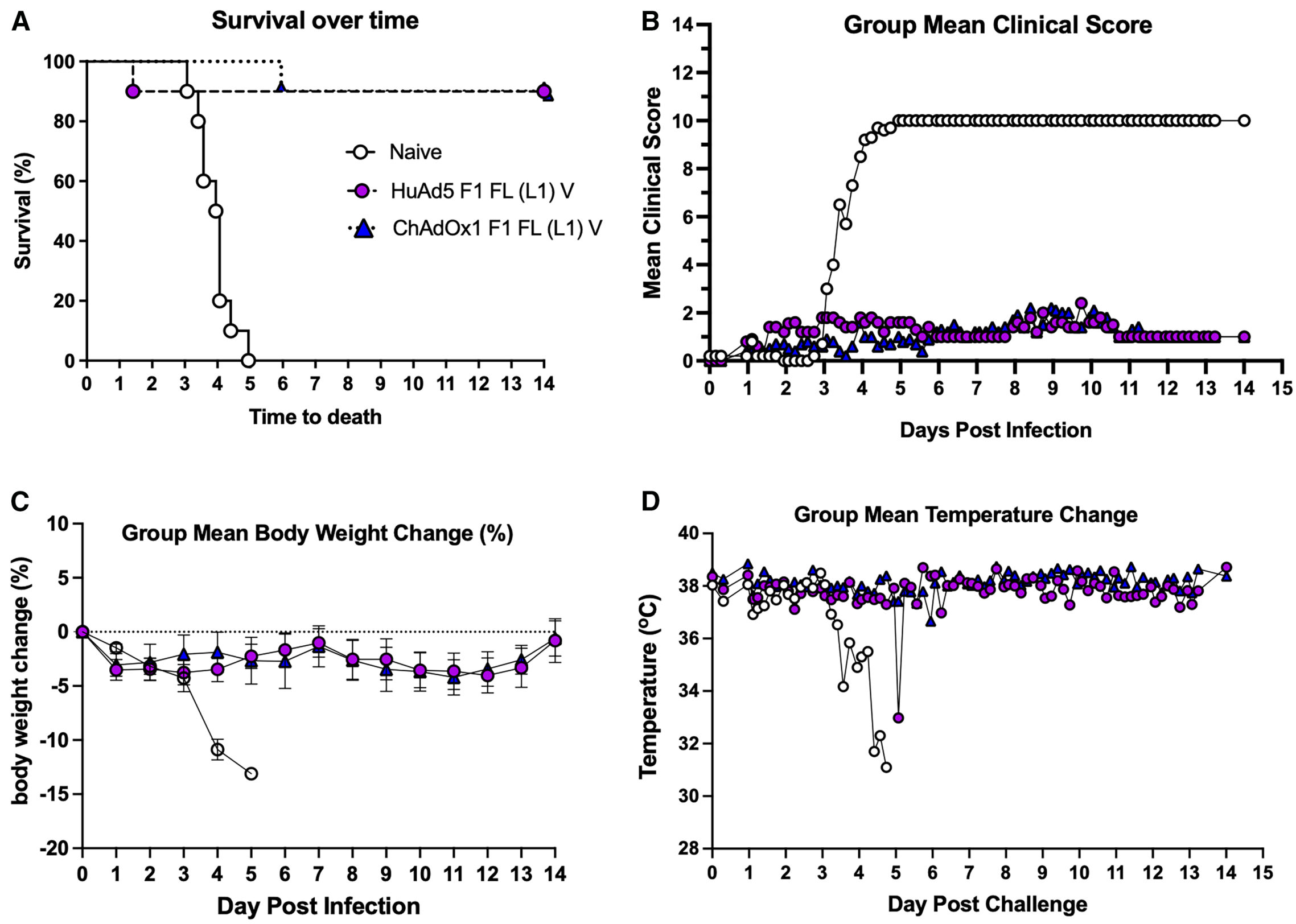

Figure 8. ChAdOx1 F1 L1 V induces similar protection against a virulent plague aerosol challenge in mice as HuAd5 F1 L1 V

Three groups of NIH Swiss mice (n = 10; 5 males and 5 females) were included in the study. One group remained naive (black), and the other groups were immunized by the intramuscular route with a single dose (5×10^8 infectious unit) of ChAdOx1 plague (F1 FL [L1] V) or HuAd5 plague (F1 FL [L1] V) (positive control). Mice were subjected to a higher dose of aerosol challenge at week 6 and were followed up for 14 days post challenge for clinical signs of illness. Shown are the survival rates at the last time point (A), the clinical scores assessed twice a day and represented over time (B), the average group surviving mouse weight over time (C), and the mean temperature change over time (D).

Stat-4 KO mice are not protected despite high antibody titers,[45] supporting the importance of T-cell response in protection against plague. Our results indicate that the number of antigen-specific IFN-γ T cells also did not correlate with the level of protection, suggesting that other T-cell readouts and exploration of antibody avidity and functionality would be more informative to identify a potential correlate of protection. In this context, it would be of interest to explore whether tissue-resident memory cells, germinal center B cells, or T follicular helper responses in spleen, lungs, lymph nodes, and liver would provide pointers to potential correlate of protection, since it has been demonstrated that lung CD4+ and CD8+ TRM cells were associated with protection after vaccination with oral Yptb1 and pulmonary infection, and that enhance germinal center T follicular helper and geminal center B cell responses correlated with robust antibody responses to plague antigens.[46,47]

Another limitation is that the intranasal route of administration did not induce the protective immunity observed with the intramuscular route, while intranasal delivery of adenoviral vaccines is immunogenic in mice,[48,49] and it was shown to induce protective responses against different pathogens in different animal models.[50,51] We showed that intramuscular and sublingual adenovirus immunization induced antigen-specific T-cell response in the lungs, but as we were unable to measure the mucosal antibody response in these experiments, no hypothesis relating to the mechanism of protection (or absence thereof) can be made. As the immunogenicity in humans seems to be weak[52] or weaker than the intramuscular injection,[53] our results thus do not support progressing the ChAdOx1 plague vaccine using this mucosal route.

These findings support the use of gene-based vaccine technologies for vaccines against *Y. pestis*. Recently, mRNA-based vaccines have demonstrated protection against plague challenge in mice.[23,24] One of these vaccines only included F1 so would be unable to protect against F1-deficient strains, but our findings support the further development of such vaccines including at least the V antigen. In this regard, an important limitation of the present study is that we did not directly evaluate the protective efficacy of the HuAd5 or ChAdOx1 F1-V vaccines against an F1-negative (acapsular) *Y. pestis* strain. Protection against F1-negative strain represents a critical stress test for plague vaccines, as immunity directed solely against the F1 capsule can be bypassed by virulent acapsular variants.

However, substantial prior work suggests that vaccines incorporating LcrV (V antigen) can confer F1-independent protection. Recombinant LcrV subunit vaccines formulated with alum have been shown to protect mice against pneumonic and bubonic plague caused by both F1-postive CO92 and F1-negative mutants,[54] while F1-V fusion protein vaccines have demonstrated protection against both F1-postive and F1-negative strains in multiple animal models.[55] Viral-vectored vaccines encoding LcrV, alone or in combination with additional antigens such as YscF, have also conferred protection against F1-negative challenge strains in murine pneumonic plague models.[56] Direct evaluation against F1-negative strains therefore represents an important priority for future challenge studies.

Finally, the ChAdOx1 plague vaccine candidate has progressed to phase I clinical evaluation in the UK and Uganda (ISRCTN4107 7863 and ISRCTN79243381), supporting its continued development as a promising countermeasure against plague.

MATERIALS AND METHODS

Study design

To investigate the potential of adenoviral-vectored vaccines against *Y. pestis*, two protective antigens were included into different designs and combinations of replication-deficient adenoviruses. Immunogenicity experiments were performed to explore the strength and longevity of the responses, as compared with a protein-based positive control (recombinant F1 and V proteins, a generous gift from Dr. Diane Williamson, Defense Science and Technology Laboratory [DSTL], UK).[52] *In vitro* expression testing in mammalian cells was performed by western blot and flow cytometry using anti-F1 (Abcam) or anti-V antibody (Invitrogen). Assessment of the immune responses was performed by quantitative ELISA and ELISpot assays. All data were included in the analysis. Sample size was determined by previous experience with preclinical vaccine development; the number of animals per group is indicated in the figures and supplemental data. Each animal was allocated randomly to a treatment group by the animal caretaker. The experimenter was not involved in the randomization. Assays included three technical replicates.

Vaccine construction and production

Sequences for F1 (NC_003134.1) and V (NC_003131.1) from *Y. pestis* CO93 strains were obtained from the NCBI sequence database. Nine transgenes were designed, including either the *caf1* or the *lcrV* genes alone or both inserted into a single vaccine vector to generate a fusion protein. Differences in the bacterial sequence used in addition to the mammalian tissue plasminogen activator signal sequence were explored,[28] and different flexible linkers between the two antigens were used (Table S1). The codon-optimized sequences were inserted into recombinant adenoviruses (human adenovirus serotype 5 and chimpanzee Oxford 1, ChAdOx1) as described previously using a Gateway-compatible entry vector.[35,57] An empty adenoviral vector and one expressing an irrelevant antigen (meningococcal group B fHbp[27]) were used as negative controls. Although adenoviral vectors are dosed as viral particles in humans, the antigen-specific immunogenicity is due to infectious virus (IU, quantified by titration) that leads to transgene expression as opposed to viral particles, which also measures non-infectious virus. Therefore, dosing as IUs was selected for these preclinical studies using HuAd5 and ChAdOx1, and the P:I ratios (particles to infectivity) were measured for all batches as described previously.[27] The recombinant proteins rF1 and V used for immunization were produced and provided by the DSTL (Porton Down, United Kingdom), and they were shown to be safe, immunogenic, and protective in mice when formulated in aluminum adjuvant and administered as a two-dose intramuscular schedule.[12] The protein profile of the recombinant F1 protein was verified by SDS PAGE and western blotting and was similar to the original description (Figure S5).

Immunogenicity experiments in mice

Procedures were performed according to the U.K. Animals (Scientific Procedures) Act 1986 and were approved by the University of Oxford Animal Care and Ethical Review Committee. Female outbred NIH Swiss or CD1 outbred mice (Harlan, UK) were housed in specific pathogen-free conditions. Negative control groups consisted of non-immunized, naive mice and/or mice immunized with an empty adenovirus experiment as indicated in the corresponding figures. Vaccines were administered via the IM or mucosal (intranasal or SL) route as indicated in the corresponding figures. The animals were bled at different time points (weeks 2, 6, 10, 18, and 26). Blood was collected from tail bleeds or terminal cardiac bleeds, allowed to clot, and centrifuged at 15,000 $\times$ *g* for 10 min. Spleens and lungs were harvested following cervical dislocation under terminal sedation.

Virulent challenge with *Y. pestis* in mice

All procedures related to the animal challenge were conducted at UKHSA Porton Down within biosafety level 3 containment (BSL3) under the authority and in compliance with a UK Home Office Project license reviewed and approved by the UKHSA Animals Scientific Procedures Ethics committee. Groups of mice immunized with Ad5 vectors were given a single dose, and the group that received the positive control (recombinant F1 and V formulated in aluminum adjuvant) received a second dose at 3 weeks. Naive mice were not immunized. Following batch propagation of *Y. pestis* CO92 (BEI, Research repository) in liquid TSB media at 26°C,[44] the bacterial inoculum aerosolization process was conducted using a Biaera digital controller connected to an adapted Henderson apparatus. Particle size, humidity, flow rate, and exposure time were monitored and recorded for each aerosol exposure procedure.[44] 6 weeks after priming, mice were loaded into Perspex restraint tubes, and groups of up to twenty mice were simultaneously placed into a flow of air containing aerosolized *Y. pestis* CO92 at an estimated dose of 6.5 MLD50 (3.25E+04 CFU). The inhaled dose was estimated by back-titration of the viable content of the inoculum impinger samples (mean 4.60E+08 CFU/mL), the estimated inhaled volume based on mean body weight (269 mL), and the estimated spray factor (2.64E−07). The survival and health of every mouse were monitored throughout the critical phase of the experiment by manual observation of every mouse at least four times a day in the first week post-infection and recordings of any clinical signs of infection. Body

weights were determined and manually recorded daily. A baseline weight for each animal was calculated from the average of weights on 1-day pre-challenge and day of challenge. These were used to calculate percent weight change from baseline at each time point. Clinical signs of infection were listed and scores assigned to each. Mice were euthanized if found to reach pre-determined endpoints, which included neurological disturbance or lack of mobility. If a mouse was found to reach a euthanasia endpoint, this was recorded (score 9) and triggered rapid euthanasia. Following euthanasia, a clinical score of 10 was assigned to the individual record and carried forward in the group scoring to assist in the graphical summary of clinical signs of infection in a semi-quantitative way over time.

Detection of antibodies by ELISA against recombinant proteins

Recombinant F1 and V proteins were diluted in PBS at 2 μg/mL, and 100 μL per well was used to coat Nunc Maxisorp ELISA plates overnight at 4°C. Plates were blocked with the assay diluent (1% BSA in PBS). Each serum sample was tested at four serial 2× dilutions in duplicate and incubated overnight at 4°C. Plates were washed five times with PBS Tween 0.05% following each incubation. The antibody response was detected with a horseradish-peroxidase-conjugated goat anti-mouse IgG (Jackson ImmunoResearch) diluted at 1:10,000 and visualized with 3,3′,5,5′-Tetramethylbensidine substrate (Sigma-Aldrich). The enzymatic reaction was stopped using 1 M H2SO4, and each sample was assigned an arbitrary unit value based on a standard curve generated on each plate using the relevant monoclonal antibody (anti-F1, Abcam ab20833, anti-F1 antigen antibody mAb [YPF19]) and anti-V antibody (Invitrogen, MA1-23088).

Statistics

Antibody titers as measured by ELISA are presented as median ±95% confidence intervals. Statistical analysis of differences between antibody titers was performed using either Kruskal-Wallis test, Mann-Whitney test, two-way ANOVA with Bonferroni post-tests, or one-way ANOVA with Dunn's multiple comparisons test when appropriate and as stated, using Prism 5 (GraphPad). The experimental units are single animals. No data exclusion was done. Potential confounders were minimized by changing orders of treatments and measurements and random cage allocation.

DATA AND CODE AVAILABILITY

All data are available in the main text or the supplemental materials.

ACKNOWLEDGMENTS

The authors thank Diane Williamson, Defense Science and Technology Laboratory, DSTL, Porton Down, Salisbury, Wiltshire, United Kingdom for the generous gift of recombinant F1 and V proteins, as well as for expert advice; the Viral Vector Core Facility (VVCF) for production and quality control of all adenovirus vaccine candidates; and the staff at the Functional Genomics Facility. A.J.P., A.V.H., and C.R. are Jenner Investigators. C.R. is supported by the Equal Opportunities Foundation (Hong Kong) and the Braithwaite Family Foundation. Y.C.K. is supported by the Wellcome Trust Grant (224117/Z/21/Z). This work was supported by a grant from Innovate UK ref. 972229 (A.J.P. and C.R.) and by the NIHR Oxford Biomedical Research Centre, Oxford, United Kingdom, Vaccine theme (A.J.P.).

AUTHOR CONTRIBUTIONS

Conceptualization: C.R., A.J.P., and A.V.S.H. Methodology: C.R., C.D., A.J.P., S.G.F., P.B., and Y.C.K. Investigation: C.D., L.S.-R., L.B., P.C., A.L., A.B.W., M.K., C.L.-C., and Y.C.K. for cloning, *in vitro* experiments, and immunogenicity in mice including all readouts; S.F., G.H., and S.G.F. performed the challenge study. Visualization: C.D. and Y.C.K. Funding acquisition: C.R. and A.J.P. Project administration: C.R. Supervision: C.R., C.D., A.J.P., S.G.F., and Y.C.K. Writing – original draft: C.R., C.D., and Y.C.K. Writing – review & editing: all.

DECLARATION OF INTERESTS

A.J.P. is Chair of U.K. Dept. Health and Social Care's (DHSC) Joint Committee on Vaccination & Immunisation (JCVI). The views expressed in this article do not necessarily represent the views of DHSC, JCVI. A.V.S.H. is named as an inventor on a patent covering use of particular simian adenoviral-vectored vaccines. P.T.B. has performed paid consultancy work for Pfizer and Sanofi Pasteur. C.R. has performed paid consultancy for Guidepoint. S.G.F. has performed paid consultancy work by the WHO. C.D., A.V.S.H., A.J.P., and C.R. are contributors to intellectual property licensed by Oxford University Innovation to AstraZeneca limited to COVID-19 applications. A.J.P. waives his rights under any patent.

SUPPLEMENTAL INFORMATION

Supplemental information can be found online at https://doi.org/10.1016/j.ymthe.2026.02.036.

REFERENCES

1. Vallès, X., Stenseth, N.C., Demeure, C., Horby, P., Mead, P.S., Cabanillas, O., Ratsitorahina, M., Rajerison, M., Andrianaivoarimanana, V., Ramasindrazana, B., et al. (2020). Human plague: An old scourge that needs new answers. PLoS Negl. Trop. Dis. *14*, e0008251. https://doi.org/10.1371/journal.pntd.0008251.
2. Ansari, I., Grier, G., and Byers, M. (2020). Deliberate release: Plague - A review. J. Biosaf. Biosecur. *2*, 10–22. https://doi.org/10.1016/j.jobb.2020.02.001.
3. Mahmoudi, A., Kryštufek, B., Sludsky, A., Schmid, B.V., DE Almeida, A.M.P., Lei, X., Ramasindrazana, B., Bertherat, E., Yeszhanov, A., Stenseth, N.C., and Mostafavi, E. (2021). Plague reservoir species throughout the world. Integr. Zool. *16*, 820–833. https://doi.org/10.1111/1749-4877.12511.
4. Abedi, A.A., Shako, J.-C., Gaudart, J., Sudre, B., Ilunga, B.K., Shamamba, S.K.B., Diatta, G., Davoust, B., Tamfum, J.-J.M., Piarroux, R., and Piarroux, M. (2018). Ecologic Features of Plague Outbreak Areas, Democratic Republic of the Congo, 2004-2014. Emerg. Infect. Dis. *24*, 210–220. https://doi.org/10.3201/eid2402.160122.
5. Apangu, T., Acayo, S., Atiku, L.A., Apio, H., Candini, G., Okoth, F., Basabose, J.K., Ojosia, L., Ajoga, S., Mongiba, G., et al. (2020). Intervention To Stop Transmission of Imported Pneumonic Plague - Uganda, 2019. MMWR Morb. Mortal. Wkly. Rep. *69*, 241–244. https://doi.org/10.15585/mmwr.mm6909a5.
6. Carlson, C.J., Bevins, S.N., and Schmid, B.V. (2022). Plague risk in the western United States over seven decades of environmental change. Glob. Chang. Biol. *28*, 753–769. https://doi.org/10.1111/gcb.15966.
7. Butler, T. (2023). Plague Gives Surprises in the Second Decade of the Twenty-First Century. Am. J. Trop. Med. Hyg. *109*, 985–988. https://doi.org/10.4269/ajtmh.23-0331.
8. Randremanana, R., Andrianaivoarimanana, V., Nikolay, B., Ramasindrazana, B., Paireau, J., Ten Bosch, Q.A., Rakotondramanga, J.M., Rahajandraibe, S., Rahelinirina, S., Rakotomanana, F., et al. (2019). Epidemiological characteristics of an urban plague epidemic in Madagascar, August-November, 2017: an outbreak report. Lancet Infect. Dis. *19*, 537–545. https://doi.org/10.1016/S1473-3099(18)30730-8.
9. Aborode, A.T., Dos Santos Costa, A.C., Mohan, A., Goyal, S., Rabiu, A.T., Tsagkaris, C., Uwishema, O., Outani, O., Ahmad, S., and Essar, M.Y. (2021). Epidemic of plague amidst COVID-19 in Madagascar: efforts, challenges, and recommendations. Trop. Med. Health *49*, 56. https://doi.org/10.1186/s41182-021-00349-5.
10. Andrianaivoarimanana, V., Wagner, D.M., Birdsell, D.N., Nikolay, B., Rakotoarimanana, F., Randriantseheno, L.N., Vogler, A.J., Sahl, J.W., Hall, C.M., Somprasong, N., et al. (2022). Transmission of Antimicrobial Resistant Yersinia pestis During a Pneumonic Plague Outbreak. Clin. Infect. Dis. *74*, 695–702. https://doi.org/10.1093/cid/ciab606.

11. Wang, X., Zhang, X., Zhou, D., and Yang, R. (2013). Live-attenuated Yersinia pestis vaccines. Expert Rev. Vaccin. *12*, 677–686. https://doi.org/10.1586/erv.13.42.

12. Williamson, E.D., Flick-Smith, H.C., Waters, E., Miller, J., Hodgson, I., Le Butt, C.S., and Hill, J. (2007). Immunogenicity of the rF1+rV vaccine for plague with identification of potential immune correlates. Microb. Pathog. *42*, 11–21. https://doi.org/10.1016/j.micpath.2006.09.003.

13. Chichester, J.A., Musiychuk, K., Farrance, C.E., Mett, V., Lyons, J., Mett, V., and Yusibov, V. (2009). A single component two-valent LcrV-F1 vaccine protects non-human primates against pneumonic plague. Vaccine *27*, 3471–3474. https://doi.org/10.1016/j.vaccine.2009.01.050.

14. Williamson, E.D., and Oyston, P.C.F. (2013). Protecting against plague: towards a next-generation vaccine. Clin. Exp. Immunol. *172*, 1–8. https://doi.org/10.1111/cei.12044.

15. Smiley, S.T. (2008). Immune defense against pneumonic plague. Immunol. Rev. *225*, 256–271. https://doi.org/10.1111/j.1600-065X.2008.00674.x.

16. Tatsis, N., and Ertl, H.C.J. (2004). Adenoviruses as vaccine vectors. Mol. Ther. *10*, 616–629. https://doi.org/10.1016/j.ymthe.2004.07.013.

17. Ewer, K.J., Lambe, T., Rollier, C.S., Spencer, A.J., Hill, A.V., and Dorrell, L. (2016). Viral vectors as vaccine platforms: from immunogenicity to impact. Curr. Opin. Immunol. *41*, 47–54. https://doi.org/10.1016/j.coi.2016.05.014.

18. Milligan, I.D., Gibani, M.M., Sewell, R., Clutterbuck, E.A., Campbell, D., Plested, E., Nuthall, E., Voysey, M., Silva-Reyes, L., McElrath, M.J., et al. (2016). Safety and Immunogenicity of Novel Adenovirus Type 26- and Modified Vaccinia Ankara-Vectored Ebola Vaccines: A Randomized Clinical Trial. JAMA *315*, 1610–1623. https://doi.org/10.1001/jama.2016.4218.

19. Barrett, J.R., Belij-Rammerstorfer, S., Dold, C., Ewer, K.J., Folegatti, P.M., Gilbride, C., Halkerston, R., Hill, J., Jenkin, D., Stockdale, L., et al. (2021). Phase 1/2 trial of SARS-CoV-2 vaccine ChAdOx1 nCoV-19 with a booster dose induces multifunctional antibody responses. Nat. Med. *27*, 279–288. https://doi.org/10.1038/s41591-020-01179-4.

20. Sadoff, J., Gray, G., Vandebosch, A., Cárdenas, V., Shukarev, G., Grinsztejn, B., Goepfert, P.A., Truyers, C., Fennema, H., Spiessens, B., et al. (2021). Safety and Efficacy of Single-Dose Ad26.COV2.S Vaccine against Covid-19. N. Engl. J. Med. *384*, 2187–2201. https://doi.org/10.1056/NEJMoa2101544.

21. Marsay, L., Dold, C., Paterson, G.K., Yamaguchi, Y., Derrick, J.P., Chan, H., Feavers, I.M., Maiden, M.C.J., Wyllie, D., Hill, A.V., et al. (2022). Viral vectors expressing group B meningococcal outer membrane proteins induce strong antibody responses but fail to induce functional bactericidal activity. J. Infect. *84*, 658–667. https://doi.org/10.1016/j.jinf.2022.02.032.

22. Rocke, T.E., Kingstad-Bakke, B., Berlier, W., and Osorio, J.E. (2014). A Recombinant Raccoon Poxvirus Vaccine Expressing both Yersinia pestis F1 and Truncated V Antigens Protects Animals against Lethal Plague. Vaccines *2*, 772–784. https://doi.org/10.3390/vaccines2040772.

23. Kon, E., Levy, Y., Elia, U., Cohen, H., Hazan-Halevy, I., Aftalion, M., Ezra, A., Bar-Haim, E., Naidu, G.S., Diesendruck, Y., et al. (2023). A single-dose F1-based mRNA-LNP vaccine provides protection against the lethal plague bacterium. Sci. Adv. *9*, eadg1036. https://doi.org/10.1126/sciadv.adg1036.

24. Shattock, R.J., Andrianaivoarimanana, V., McKay, P.F., Randriantseheno, L.N., Murugaiah, V., Samnuan, K., Rogers, P., Tregoning, J.S., Rajerison, M., Moore, K.M., et al. (2023). A self-amplifying RNA vaccine provides protection in a murine model of bubonic plague. Front. Microbiol. *14*, 1247041.

25. Chiuchiolo, M.J., Boyer, J.L., Krause, A., Senina, S., Hackett, N.R., and Crystal, R.G. (2006). Protective immunity against respiratory tract challenge with Yersinia pestis in mice immunized with an adenovirus-based vaccine vector expressing V antigen. J. Infect. Dis. *194*, 1249–1257. https://doi.org/10.1086/507644.

26. Sha, J., Kirtley, M.L., Klages, C., Erova, T.E., Telepnev, M., Ponnusamy, D., Fitts, E.C., Baze, W.B., Sivasubramani, S.K., Lawrence, W.S., et al. (2016). A Replication-Defective Human Type 5 Adenovirus-Based Trivalent Vaccine Confers Complete Protection against Plague in Mice and Nonhuman Primates. Clin. Vaccin. Immunol. *23*, 586–600. https://doi.org/10.1128/CVI.00150-16.

27. Dold, C., Marsay, L., Wang, N., Silva-Reyes, L., Clutterbuck, E., Paterson, G.K., Sharkey, K., Wyllie, D., Beernink, P.T., Hill, A.V., et al. (2023). An adenoviral-vectored vaccine confers seroprotection against capsular group B meningococcal disease. Sci. Transl. Med. *15*, eade3901. https://doi.org/10.1126/scitranslmed.ade3901.

28. Sheerin, D., Dold, C., Silva-Reyes, L., Linder, A., Pollard, A.J., and Rollier, C.S. (2022). Inclusion of a dual signal sequence enhances the immunogenicity of a novel viral vectored vaccine against the capsular group B meningococcus. Cell Biosci. *12*, 86. https://doi.org/10.1186/s13578-022-00809-3.

29. Folegatti, P.M., Harrison, K., Preciado-Llanes, L., Lopez, F.R., Bittaye, M., Kim, Y.C., Flaxman, A., Bellamy, D., Makinson, R., Sheridan, J., et al. (2021). A single dose of ChAdOx1 Chik vaccine induces neutralizing antibodies against four chikungunya virus lineages in a phase 1 clinical trial. Nat. Commun. *12*, 4636. https://doi.org/10.1038/s41467-021-24906-y.

30. Gomi, R., Sharma, A., Wu, W., Sung, B., and Worgall, S. (2017). Post-exposure immunization by capsid-modified AdC7 vector expressing Pseudomonas aeruginosa OprF clears P. aeruginosa respiratory infection. Vaccine *35*, 7174–7180. https://doi.org/10.1016/j.vaccine.2017.10.078.

31. Koroleva, E.A., Kobets, N.V., Shcherbinin, D.N., Zigangirova, N.A., Shmarov, M.M., Tukhvatulin, A.I., Logunov, D.Y., Naroditsky, B.S., and Gintsburg, A.L. (2017). Chlamydial Type III Secretion System Needle Protein Induces Protective Immunity against Chlamydia muridarum Intravaginal Infection. Biomed. Res. Int. *2017*, 3865802. https://doi.org/10.1155/2017/3865802.

32. Wang, J., Thorson, L., Stokes, R.W., Santosuosso, M., Huygen, K., Zganiacz, A., Hitt, M., and Xing, Z. (2004). Single mucosal, but not parenteral, immunization with recombinant adenoviral-based vaccine provides potent protection from pulmonary tuberculosis. J. Immunol. *173*, 6357–6365. https://doi.org/10.4049/jimmunol.173.10.6357.

33. Heinz, F.X., and Stiasny, K. (2021). Distinguishing features of current COVID-19 vaccines: knowns and unknowns of antigen presentation and modes of action. NPJ Vaccin. *6*, 104. https://doi.org/10.1038/s41541-021-00369-6.

34. Tapia, M.D., Sow, S.O., Mbaye, K.D., Thiongane, A., Ndiaye, B.P., Ndour, C.T., Mboup, S., Keshinro, B., Kinge, T.N., Vernet, G., et al. (2020). Safety, reactogenicity, and immunogenicity of a chimpanzee adenovirus vectored Ebola vaccine in children in Africa: a randomised, observer-blind, placebo-controlled, phase 2 trial. Lancet Infect. Dis. *20*, 719–730. https://doi.org/10.1016/S1473-3099(20)30019-0.

35. Folegatti, P.M., Ewer, K.J., Aley, P.K., Angus, B., Becker, S., Belij-Rammerstorfer, S., Bellamy, D., Bibi, S., Bittaye, M., Clutterbuck, E.A., et al. (2020). Safety and immunogenicity of the ChAdOx1 nCoV-19 vaccine against SARS-CoV-2: a preliminary report of a phase 1/2, single-blind, randomised controlled trial. Lancet Lond. Engl. *396*, 467–478. https://doi.org/10.1016/S0140-6736(20)31604-4.

36. Williamson, E.D., Eley, S.M., Griffin, K.F., Green, M., Russell, P., Leary, S.E., Oyston, P.C., Easterbrook, T., Reddin, K.M., and Robinson, A. (1995). A new improved sub-unit vaccine for plague: the basis of protection. FEMS Immunol. Med. Microbiol. *12*, 223–230. https://doi.org/10.1111/j.1574-695X.1995.tb00196.x.

37. Rocke, T.E., Iams, K.P., Dawe, S., Smith, S.R., Williamson, J.L., Heisey, D.M., and Osorio, J.E. (2009). Further development of raccoon poxvirus-vectored vaccines against plague (Yersinia pestis). Vaccine *28*, 338–344. https://doi.org/10.1016/j.vaccine.2009.10.043.

38. Rocke, T.E., Pussini, N., Smith, S.R., Williamson, J., Powell, B., and Osorio, J.E. (2010). Consumption of baits containing raccoon pox-based plague vaccines protects black-tailed prairie dogs (Cynomys ludovicianus). Vector Borne Zoonotic Dis. *10*, 53–58. https://doi.org/10.1089/vbz.2009.0050.

39. Jones, S.M., Griffin, K.F., Hodgson, I., and Williamson, E.D. (2003). Protective efficacy of a fully recombinant plague vaccine in the guinea pig. Vaccine *21*, 3912–3918. https://doi.org/10.1016/s0264-410x(03)00379-7.

40. Miller, J., Williamson, E.D., Lakey, J.H., Pearce, M.J., Jones, S.M., and Titball, R.W. (1998). Macromolecular organisation of recombinant Yersinia pestis F1 antigen and the effect of structure on immunogenicity. FEMS Immunol. Med. Microbiol. *21*, 213–221. https://doi.org/10.1111/j.1574-695X.1998.tb01168.x.

41. Mizel, S.B., Graff, A.H., Sriranganathan, N., Ervin, S., Lees, C.J., Lively, M.O., Hantgan, R.R., Thomas, M.J., Wood, J., and Bell, B. (2009). Flagellin-F1-V Fusion Protein Is an Effective Plague Vaccine in Mice and Two Species of Nonhuman Primates. Clin. Vaccin. Immunol. *16*, 21–28. https://doi.org/10.1128/CVI.00333-08.

42. Williamson, E.D. (2012). The role of immune correlates and surrogate markers in the development of vaccines and immunotherapies for plague. Adv. Prev. Med. *2012*, 365980. https://doi.org/10.1155/2012/365980.

43. Hill, J., Copse, C., Leary, S., Stagg, A.J., Williamson, E.D., and Titball, R.W. (2003). Synergistic protection of mice against plague with monoclonal antibodies specific for the F1 and V antigens of Yersinia pestis. Infect. Immun. *71*, 2234–2238. https://doi.org/10.1128/IAI.71.4.2234-2238.2003.

44. Graham, V.A., Hatch, G.J., Bewley, K.R., Steeds, K., Lansley, A., Bate, S.R., and Funnell, S.G.P. (2014). Efficacy of Primate Humoral Passive Transfer in a Murine Model of Pneumonic Plague Is Mouse Strain-Dependent. J. Immunol. Res. *2014*, 807564. https://doi.org/10.1155/2014/807564.

45. Elvin, S.J., and Williamson, E.D. (2004). Stat 4 but not Stat 6 mediated immune mechanisms are essential in protection against plague. Microb. Pathog. *37*, 177–184. https://doi.org/10.1016/j.micpath.2004.06.009.

46. Singh, A.K., Majumder, S., Wang, X., Song, R., and Sun, W. (2023). Lung Resident Memory T Cells Activated by Oral Vaccination Afford Comprehensive Protection against Pneumonic *Yersinia pestis* Infection. J. Immunol. *210*, 259–270. https://doi.org/10.4049/jimmunol.2200487.

47. Galloway, D.R., Nguyen, N.X., Li, J., Houston, N., Gregersen, G., Williamson, E.D., Falkenberg, F.W., Herron, J.N., and Hale, J.S. (2022). The magnitude of the germinal center B cell and T follicular helper cell response predicts long-lasting antibody titers to plague vaccination. Front. Immunol. *13*, 1017385. https://doi.org/10.3389/fimmu.2022.1017385.

48. Croyle, M.A., Patel, A., Tran, K.N., Gray, M., Zhang, Y., Strong, J.E., Feldmann, H., and Kobinger, G.P. (2008). Nasal delivery of an adenovirus-based vaccine bypasses pre-existing immunity to the vaccine carrier and improves the immune response in mice. PLoS One *3*, e3548. https://doi.org/10.1371/journal.pone.0003548.

49. Freitag, T.L., Fagerlund, R., Karam, N.L., Leppänen, V.-M., Ugurlu, H., Kant, R., Mäkinen, P., Tawfek, A., Jha, S.K., Strandin, T., et al. (2023). Intranasal administration of adenoviral vaccines expressing SARS-CoV-2 spike protein improves vaccine immunity in mouse models. Vaccine *41*, 3233–3246. https://doi.org/10.1016/j.vaccine.2023.04.020.

50. Bricker, T.L., Darling, T.L., Hassan, A.O., Harastani, H.H., Soung, A., Jiang, X., Dai, Y.-N., Zhao, H., Adams, L.J., Holtzman, M.J., et al. (2021). A single intranasal or intramuscular immunization with chimpanzee adenovirus-vectored SARS-CoV-2 vaccine protects against pneumonia in hamsters. Cell Rep. *36*, 109400. https://doi.org/10.1016/j.celrep.2021.109400.

51. Afkhami, S., D'Agostino, M.R., Vaseghi-Shanjani, M., Lepard, M., Yang, J.X., Lai, R., Choi, M.W.Y., Chacon, A., Zganiacz, A., Franken, K.L.M.C., et al. (2023). Intranasal multivalent adenoviral-vectored vaccine protects against replicating and dormant M.tb in conventional and humanized mice. NPJ Vaccin. *8*, 25. https://doi.org/10.1038/s41541-023-00623-z.

52. Madhavan, M., Ritchie, A.J., Aboagye, J., Jenkin, D., Provstgaad-Morys, S., Tarbet, I., Woods, D., Davies, S., Baker, M., Platt, A., et al. (2022). Tolerability and immunogenicity of an intranasally-administered adenovirus-vectored COVID-19 vaccine: An open-label partially-randomised ascending dose phase I trial. eBioMedicine *85*, 104298. https://doi.org/10.1016/j.ebiom.2022.104298.

53. Green, C.A., Scarselli, E., Sande, C.J., Thompson, A.J., de Lara, C.M., Taylor, K.S., Haworth, K., Del Sorbo, M., Angus, B., Siani, L., et al. (2015). Chimpanzee adenovirus- and MVA-vectored respiratory syncytial virus vaccine is safe and immunogenic in adults. Sci. Transl. Med. *7*, 300ra126. https://doi.org/10.1126/scitranslmed.aac5745.

54. Anderson, G.W., Leary, S.E., Williamson, E.D., Titball, R.W., Welkos, S.L., Worsham, P.L., and Friedlander, A.M. (1996). Recombinant V antigen protects mice against pneumonic and bubonic plague caused by F1-capsule-positive and -negative strains of Yersinia pestis. Infect. Immun. *64*, 4580–4585. https://doi.org/10.1128/iai.64.11.4580-4585.1996.

55. Heath, D.G., Anderson, G.W., Mauro, J.M., Welkos, S.L., Andrews, G.P., Adamovicz, J., and Friedlander, A.M. (1998). Protection against experimental bubonic and pneumonic plague by a recombinant capsular F1-V antigen fusion protein vaccine. Vaccine *16*, 1131–1137. https://doi.org/10.1016/s0264-410x(98)80110-2.

56. Kilgore, P.B., Sha, J., Andersson, J.A., Motin, V.L., and Chopra, A.K. (2021). A new generation needle- and adjuvant-free trivalent plague vaccine utilizing adenovirus-5 nanoparticle platform. Npj Vaccin. *6*, 21. https://doi.org/10.1038/s41541-020-00275-3.

57. Rollier, C.S., Spencer, A.J., Sogaard, K.C., Honeycutt, J., Furze, J., Bregu, M., Gilbert, S.C., Wyllie, D., and Hill, A.V.S. (2020). Modification of Adenovirus vaccine vector-induced immune responses by expression of a signalling molecule. Sci. Rep. *10*, 5716. https://doi.org/10.1038/s41598-020-61730-8.

YMTHE, Volume *34*

Supplemental Information

An adenoviral-vectored vaccine protects mice against aerosol challenge with *Yersinia pestis*

Christina Dold, Laura Silva-Reyes, Young Chan Kim, Luke Blackwell, Patricia Campos, Aline Linder, Alice Bridges-Webb, Mwila Kasanyinga, Cesar Lopez-Camacho, Amy Challis, Graham Hatch, Susan Fotheringham, Peter T. Beernink, Adrian V. Hill, Simon G.P. Funnell, A.J. Pollard, and C.S. Rollier

Supplemental Materials and Methods

Detection of antigen expression in cell culture

Detection of antigen expression was performed in HeLa cells infected with the antigen-expressing or control vectors as described previously *(28)*, using anti-F1 (Abcam) and V antibodies, and recombinant F1 and V proteins (generous gift from Dr Diane Williamson, DSTL, UK)*(52)*. Western blotting was performed on cell lysate and cell supernatant: Transduced HeLa cells were washed twice with ice-cold PBS and harvested in cold PBS. Cells were lysed in 1X Laemmli buffer. For supernatant, 4X Laemmli buffer was added; both cells and supernatant were boiled at 100°C for five minutes. In addition, a fraction of the supernatant was concentrated using an Amicon Ultra column with a 3kDa cut-off (Millipore, UFC200324. Concentrated samples were prepared in 4X Laemmli buffer and boiled at 100°C. Total extracts and cell supernatants (15ul volume) were resolved by SDS/PAGE and transferred to PVDF membranes. Blots were blocked with PBS-0.1% (v/v) Tween plus 5% (v/v) milk and incubated with anti-F1 (Abcam ab20833, anti-F1 antigen antibody mAb [YPF19]) or anti-V antibody (Invitrogen, MA1-23088) for one hour, followed by 5 washes with PBS- 0.1% (v/v) Tween and incubation with HRP-conjugated secondary antibody (1:5000). After final wash, Chemiluminescence (Perkin-Elmer Life Sciences, Boston, MA) was visualized using the BioRad ChemiDoc SRS device.

Splenocyte extraction

After removal, spleens were transferred to gentleMACS C tubes (Miltenyi Biotec) containing 5ml of autoMACS running buffer (Miltenyi Biotec) and dissociated using the spleen program

on the gentleMACS Dissociator (Miltenyi Biotec). After centrifugation at 300g for 10 minutes, the pellets were resuspended in Red Blood Cell Lysis solution (Qiagen) for 5 minutes. The reaction was stopped by the addition of 10ml of AutoMACS Rinsing solution (Miltenyi Biotec). After a second centrifugation at 300g for 10 minutes, cells were filtered through 70µm sieve into sterile 50ml tubes and centrifuged again. Cells were then resuspended in 10ml of DMEM+10%FBS (Sigma) and counted using a Muse Cell Analyzer counter (Luminex). Concentration was then adjusted at 4 x 106 cells/ml in DMEM+10%FBS for T cell fluorospots.

Lung cell extraction

After removal, lungs were transferred to gentleMACS C tubes containing 5ml of digestion medium (DMEM+10%FBS with 30µg/ml DNAse (Sigma) and 0.7µg/ml Collagenase (Sigma)). Lungs were kept on ice until dissociation with the gentleMACS Dissociator (lung_02 program) and then placed in a shaking incubator for 60 minutes at 37°C. Dissociated lungs were then filtered through 100µm sieve into sterile 50ml tubes. After addition of 25ml of cold DMEM+10%FBS, tubes were centrifuged at 300g for 10 minutes. Pellets were resuspended in RBC lysis buffer for 5 minutes maximum and then filtered through a 70µm sieved after addition of 10ml of cold DMEM+10%FBS. After centrifugation, cells were resuspended in 5ml DMEM+10%FBS and counted with the Muse Cell Analyzer counter (Luminex).

Detection and quantification of T-cell responses

IFN-γ and IL-5 producing T cells were quantified by using a mouse FluoroSpot kit (Mabtech, FSP-4144-10). After activation with 15 µl 35% Ethanol and 5 washes with sterile water, plates

were coated with anti-IFNγ and anti-IL-5 monoclonal capture antibodies diluted in sterile PBS at 15 μg/ml and 10 μg/ml, respectively. Plates were then sealed and kept overnight at 4 °C. The following day, plates were washed 5 times with sterile PBS and blocked with DMEM+10%FBS for minimum 30 minutes at room temperature. To ensure efficient and reproducible stimulation of antigen-specific T-cells independent of variability in antigen uptake, processing and presentation, we used overlapping peptide pools spanning the full antigen sequence of the F1 and V proteins. Two peptide pools (15-mer overlapping by 11-amino acids) were diluted in DMEM+10%FBS at 6μg/ml (final concentration after addition of cells:3μg/ml). 50μl of diluted F1 and V peptide pools were added to the wells. DMSO (Sigma) and concanavalin A (Sigma) were used as negative and positive controls for each sample. After being resuspended at 4 x 10^6 cells/ml in DMEM+10%FBS, 50μl of splenocytes or lung cells were added to the peptides wells in triplicates and incubated for 18-20h at 37 °C, 5% CO2, 95% humidity. Detection of spots was undertaken according to the manufacturer's instructions (Mabtech) and analysed with AID ELISpot software 8.0 (Autoimmun Diagnostika).

Supplemental tables:

Table S1. Characteristics of the nine transgene designs. Designs included F1 and V genes inserted into separate or a single vaccine vector, with differences in the bacterial sequence used in addition to a mammalian signal sequence, and different flexible linkers between the two antigens

Transgene	Antigens inserted	Bacterial signal sequence (SS)	Linker
F1 FL[1]	F1	Wild type SS	None
F1 T[2]	F1	No SS	None
F1 fHbpSS[3]	F1	fHbp SS	None
V	V	No SS	None
F1 T (L1) V	F1 + V	No SS	L1 [4]
F1 T (L2) V	F1 + V	No SS	L2 [5]
F1 FL (L1) V	F1 + V	Wild type SS	L1
F1 FL (L2) V	F1 + V	Wild type SS	L2
fHbpSS F1 (L1) V	F1 + V	fHbp SS	L1

[1] FL: full length immature F1 protein, containing its wild type signal sequence

[2] T: truncated mature F1 protein (no bacterial signal sequence)

[3] fHbpSS: meningococcus fHbp signal sequence NRTAFCCLSLTTALILTACSSG

[4] L1: linker used to fuse F1 and V, amino acid sequence ADLPSLAADFVE

[5] L2: linker used to fuse F1 and V, amino acid sequence GGGGSGGGGSGGGGS

Table S2. Number of mouse sera assessed by F1 ELISA at each time point in the longitudinal experiment dataset shown in Figure 2. Groups of 6 or 12 mice were immunized with the vaccines mentioned in the first column. Tail bleeds were collected at 2, 6, 10, 18, 26 and 34 weeks, without exceeding the ethically permitted maximum amount of blood per mouse, per time point and per month. Too many datapoints were missing at week 10. The number of sera collected and assessed by F1 ELISA is indicated in the table, and data shown in Figure 2B to E.

Vaccine group	N	Week 2	Week 18	Week 26	Week 34
Naive	12	8	2	11	9
rF1 + rV proteins	6	6	4	6	5
HuAd5 empty	6	6	5	5	5
HuAd5 F1 FL	6	6	6	6	5
HuAd5 F1 fHbpSS	6	6	6	6	6
HuAd5 F1 T	6	6	6	6	6
HuAd5 F1 FL (L1) V	12	11	11	10	12
HuAd5 F1 FL (L2) V	6	5	6	6	6
HuAd5 F1 fHbpSS (L1) V	6	6	6	6	6
HuAd5 F1 T (L1) V	6	5	6	6	6
HuAd5 F1 T (L2) V	6	6	5	6	6

Table S3. Number of mouse sera assessed by V ELISA at each time point in the longitudinal experiment dataset shown in Figure 2. Groups of 6 or 12 mice were immunized with the vaccines mentioned in the first column. Tail bleeds were collected at 2, 6, 10, 18, 26 and 34 weeks, without exceeding the ethically permitted maximum amount of blood per mouse, per time point and per month. Too many datapoints were missing at week 10, 26 and 34. The

number of sera collected and assessed by V ELISA is indicated in the table, and data shown in Figure 2F and G.

Vaccine group	N	Week 2	Week 18
Naive	12	12	2
rF1 + rV proteins	6	6	5
HuAd5 empty	6	5	4
HuAd5 V	6	6	6
HuAd5 F1 FL (L1) V	12	11	11
HuAd5 F1 FL (L2) V	6	6	4
HuAd5 F1 fHbpSS (L1) V	6	6	5
HuAd5 F1 T (L1) V	6	6	6
HuAd5 F1 T (L2) V	6	6	6

Supplemental Figures:

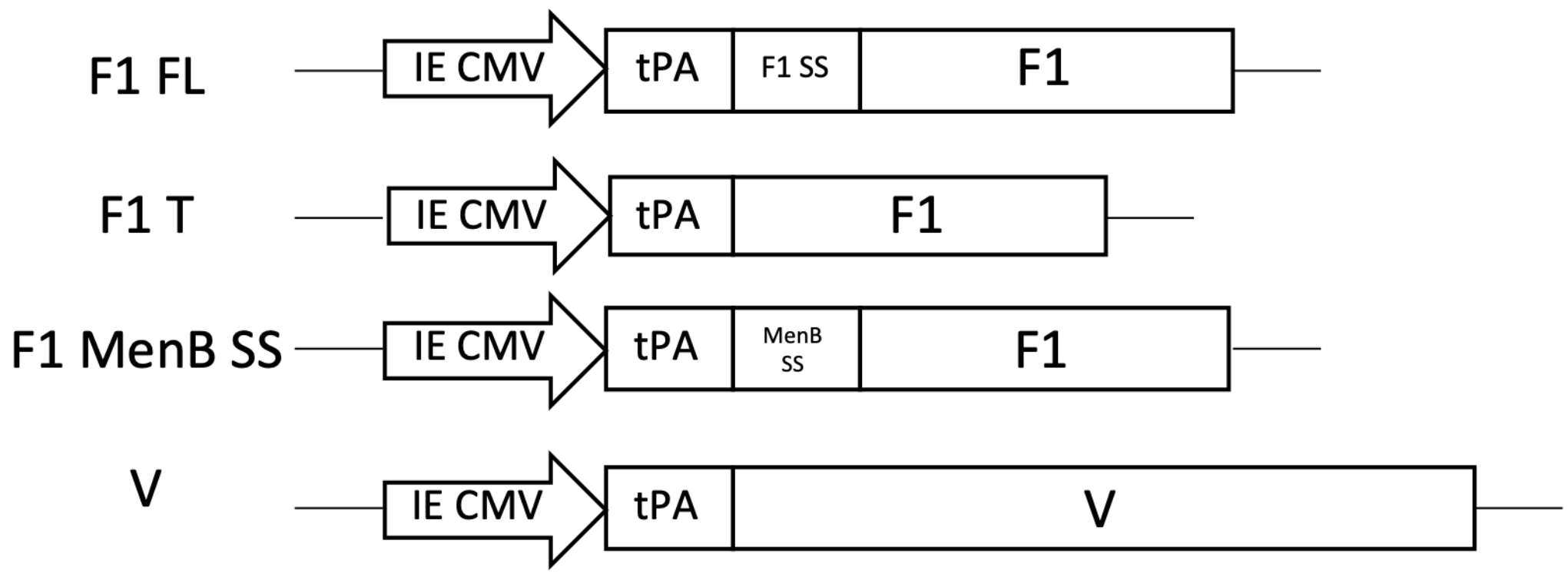

Figure S1. Schematic representation of the four monovalent transgenes, encoding either F1 or V. IE CMV: human cytomegalovirus major immediate early promoter. tPA: Human tissue plasminogen activator (tPA) signal peptide sequence. F1 signal sequence (SS): MKKISSVIAIALFG- TIATANAAD, fHbp signal sequence: NRTAFCCLSLTTALILTACSSG. The transgenes were inserted into the E1 region of the HuAd5 genome.

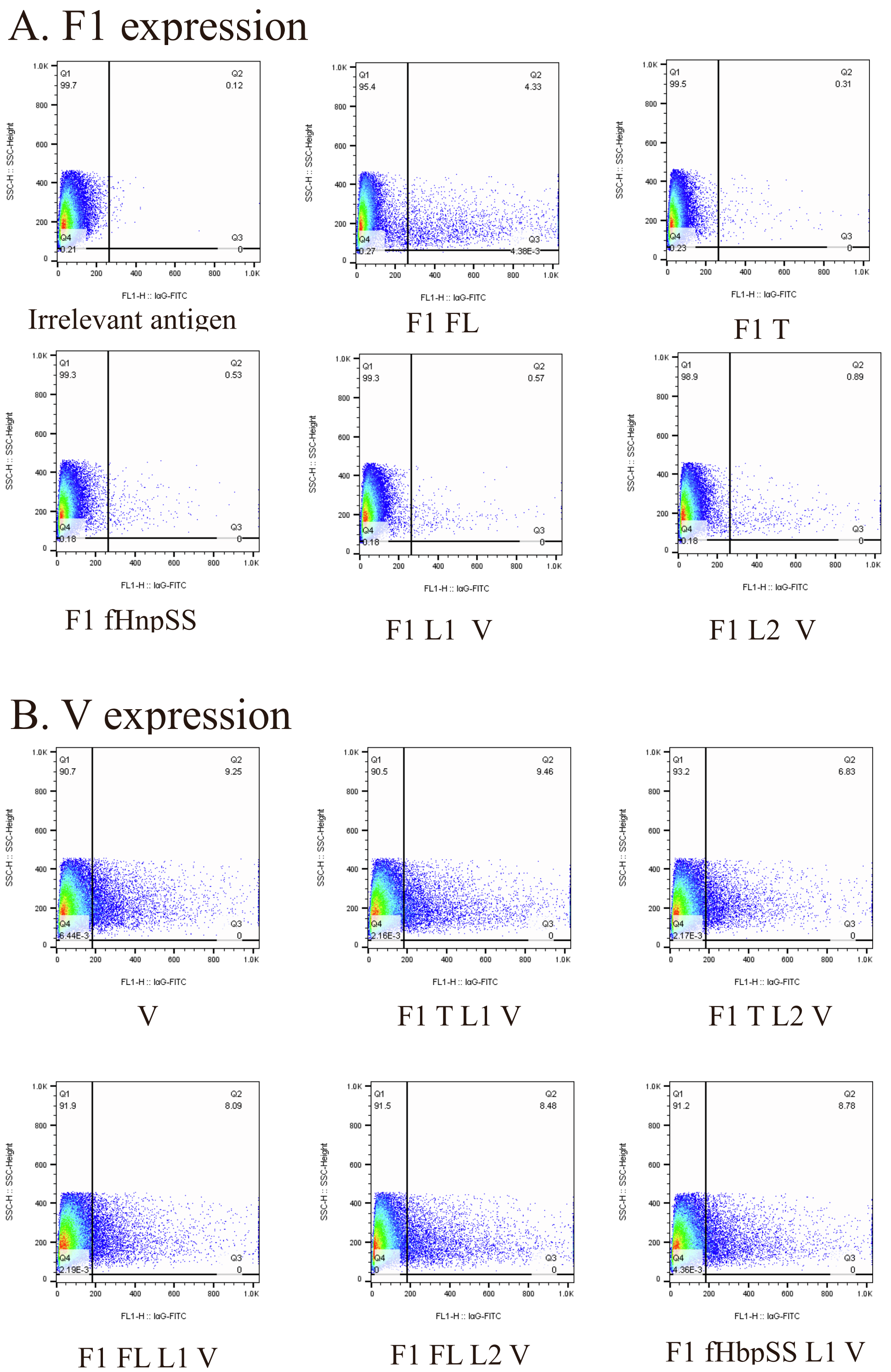

Figure S2. The different monovalent or fusion constructs express F1 (A) and V (B) in HeLa cells. Cells were infected with the HuAd5 Adenoviral vectors encoding the antigens indicated. Detection of F1 and V was performed by flow cytometry using anti-F1 and anti-V

monoclonal antibodies. Panels show the protein expression in viable cells with the constructs indicated under each panel, in one representative experiment. The F1 constructs differed in the antigen bacterial signal sequence used after the tPA signal: either full length F1 bacterial sequence (FL), no bacterial sequence (T) after the tPA, or the F1 bacterial sequence replaced with the group B meningococcal (MenB) fHbp bacterial signal sequence (fHbpSS). The fusion constructs containing both F1 and V were constructed with two different flexible linkers (L1 or L2).

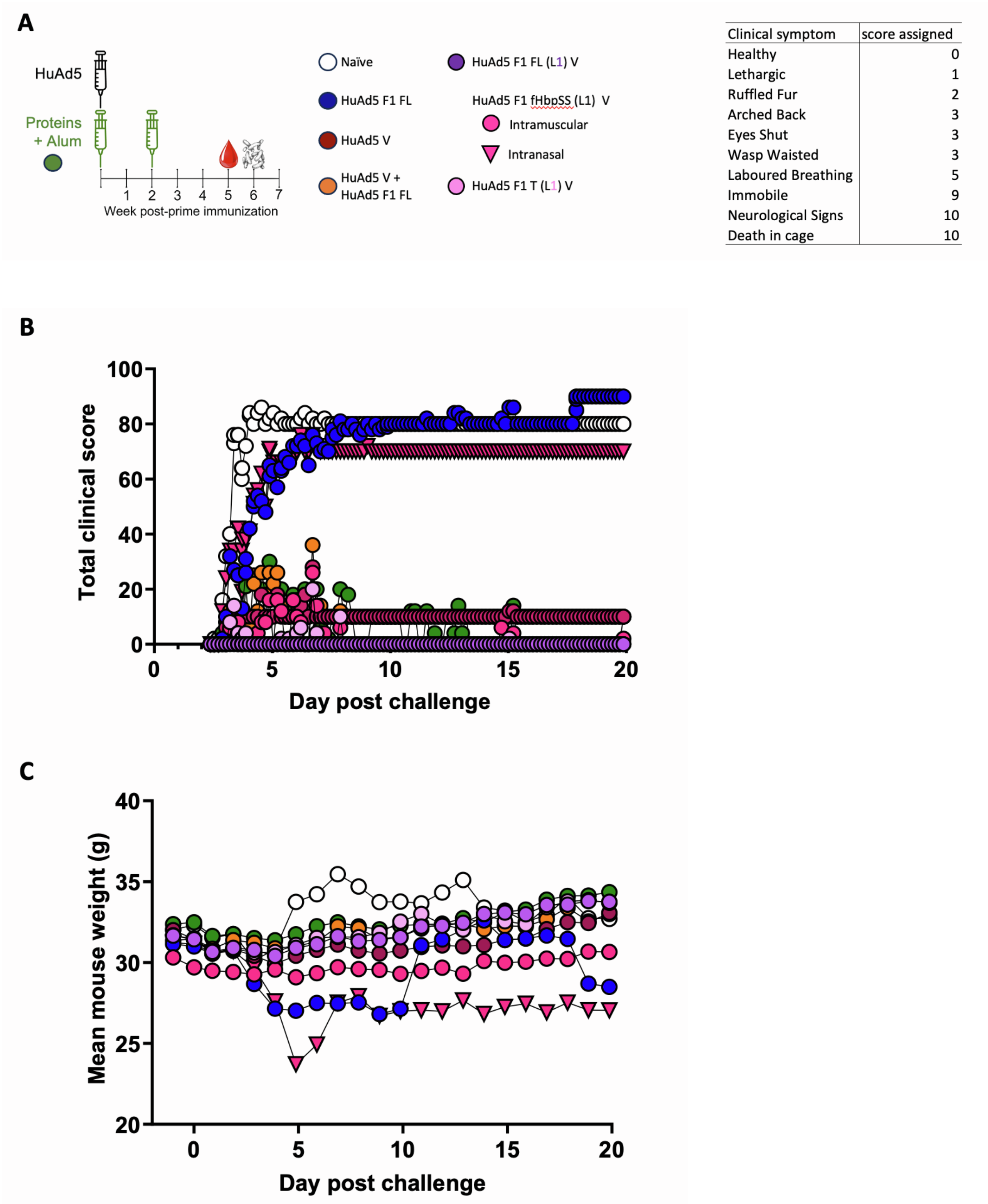

Clinical symptom	score assigned
Healthy	0
Lethargic	1
Ruffled Fur	2
Arched Back	3
Eyes Shut	3
Wasp Waisted	3
Laboured Breathing	5
Immobile	9
Neurological Signs	10
Death in cage	10

Figure S3. Impact of the different vaccines on clinical scores and mouse weights post challenge in mice. Nine groups of NIH Swiss mice (n=10) were included in the study as described in Figure 4A and panel A. All vaccines were administered by the intramuscular route

except for one group which received HuAd5 F1 fHbp SS (L1) V by the intranasal route One mouse died for unrelated reason in this group, reducing the number to 9 animals. The clinical scores described in (A) were assessed twice a day and represented over time (B). The average group surviving mouse weight over time are shown in panel C.

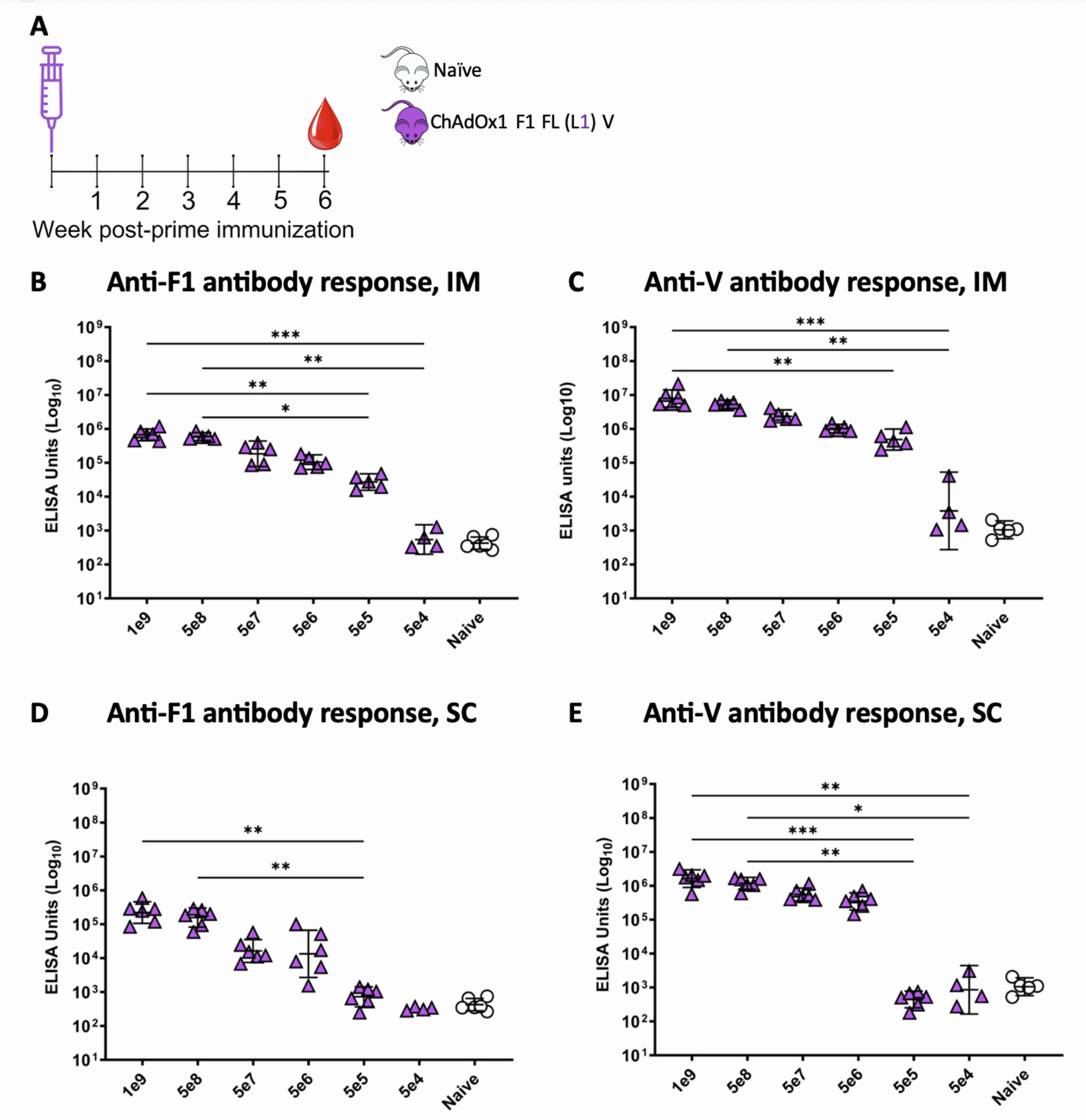

Figure S4. Dose response of the clinically relevant ChadOx1 Plague vaccine candidate given by the intramuscular (IM) and subcutaneous (SC) routes in NIH Swiss mice. Groups of outbred

NIH Swiss mice (n=6) were immunized with a single dose of ChAdOx1 (purple triangles) Plague at the doses indicated in the X axis of each panel ($5x10^4$ to 10^9 infectious units). ChAdOx1 Plague was administered by the intramuscular (IM) route as shown in panels B and C or the subcutaneous (SC) route in panels D and E. The serum IgG to F1 (B) and V (C) respectively were measured at week 6. Shown are individual responses with geometric mean and 95% confidence intervals for each group. Data were analyzed by Kruskal Wallis with Dunns multiple comparison test (* p<0.05, ** p<0.01, *** p<0.001).

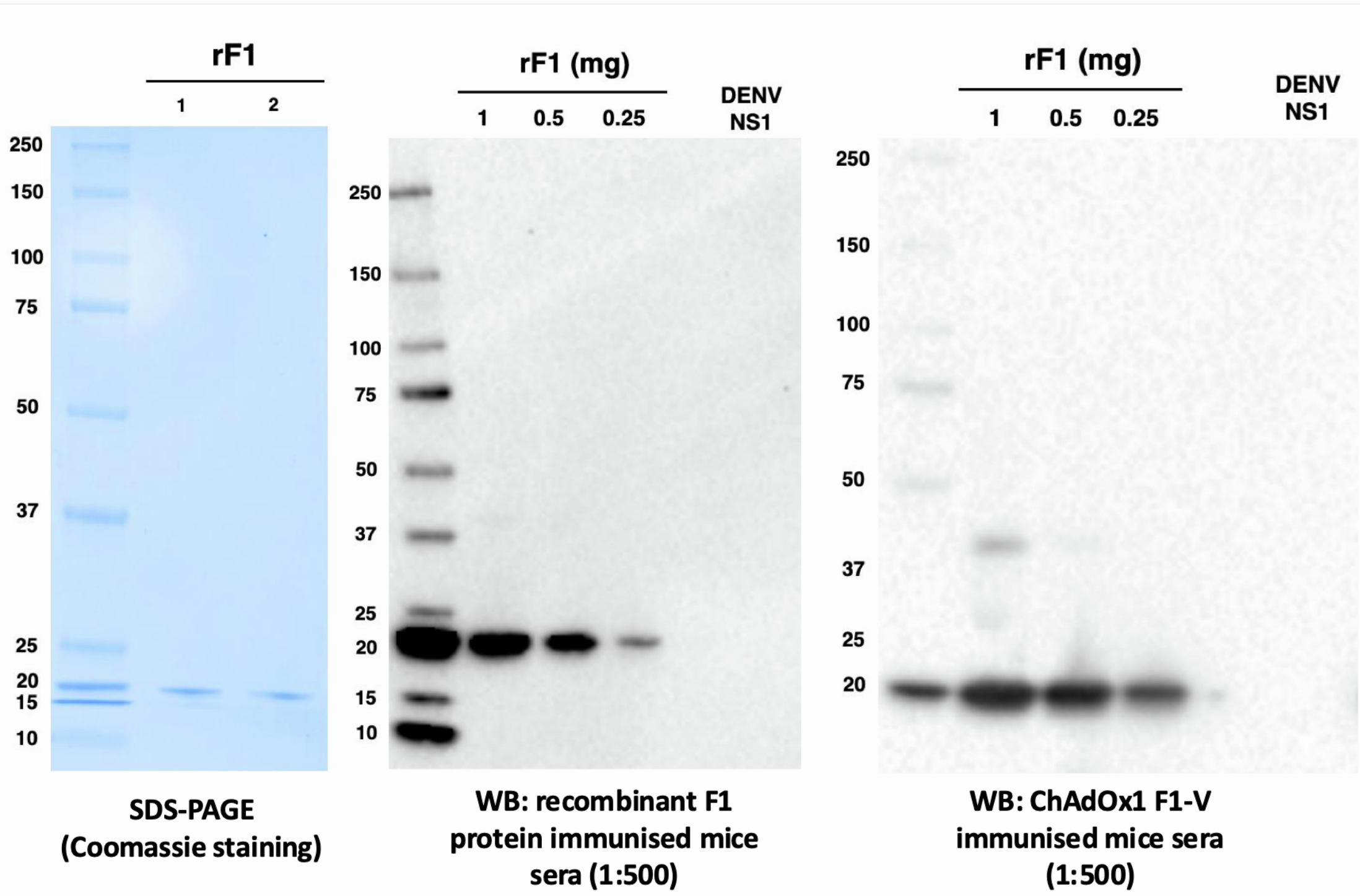

Figure S5. The recombinant F1 protein profile is similar to the original batch. SDS-PAGE (left panel) and western blot (central and right panels) of the recombinant F1 detected with the sera indicated below each panel. rF1 indicates recombinant F1, the number above the lanes indicate the loading doses, DENV NS1 is a negative control (Dengue virus non-structural protein 1), WB: western blot.
